# Spatiotemporal transcriptomic atlas reveals the dynamic characteristics and key regulators of planarian regeneration

**DOI:** 10.1038/s41467-023-39016-0

**Published:** 2023-06-02

**Authors:** Guanshen Cui, Kangning Dong, Jia-Yi Zhou, Shang Li, Ying Wu, Qinghua Han, Bofei Yao, Qunlun Shen, Yong-Liang Zhao, Ying Yang, Jun Cai, Shihua Zhang, Yun-Gui Yang

**Affiliations:** 1 CAS Key Laboratory of Genomic and Precision Medicine, Collaborative Innovation Center of Genetics and Development, College of Future Technology, Beijing Institute of Genomics, Chinese Academy of Sciences, Beijing, 100101 China; 2 China National Center for Bioinformation, Beijing, 100101 China; 3 NCMIS, CEMS, RCSDS, Academy of Mathematics and Systems Science, Chinese Academy of Sciences, Beijing, 100190 China; 4 School of Mathematical Sciences, University of Chinese Academy of Sciences, Beijing, 100049 China; 5 University of Chinese Academy of Sciences, Beijing, China; 6 Sino-Danish College, University of Chinese Academy of Sciences, Beijing, 101408 China; 7 Institute of Stem Cell and Regeneration, Chinese Academy of Sciences, Beijing, 100101 China; 8 Center for Excellence in Animal Evolution and Genetics, Chinese Academy of Sciences, Kunming, 650223 China; 9 Key Laboratory of Systems Health Science of Zhejiang Province, School of Life Science, Hangzhou Institute for Advanced Study, University of Chinese Academy of Sciences, Hangzhou, 310024 China

**Keywords:** Regeneration, Differentiation, Stem-cell differentiation

## Abstract

Whole-body regeneration of planarians is a natural wonder but how it occurs remains elusive. It requires coordinated responses from each cell in the remaining tissue with spatial awareness to regenerate new cells and missing body parts. While previous studies identified new genes essential to regeneration, a more efficient screening approach that can identify regeneration-associated genes in the spatial context is needed. Here, we present a comprehensive three-dimensional spatiotemporal transcriptomic landscape of planarian regeneration. We describe a pluripotent neoblast subtype, and show that depletion of its marker gene makes planarians more susceptible to sub-lethal radiation. Furthermore, we identified spatial gene expression modules essential for tissue development. Functional analysis of hub genes in spatial modules, such as *plk1*, shows their important roles in regeneration. Our three-dimensional transcriptomic atlas provides a powerful tool for deciphering regeneration and identifying homeostasis-related genes, and provides a publicly available online spatiotemporal analysis resource for planarian regeneration research.

## Abstract (web summary)

Cui et al. present a comprehensive three-dimensional spatiotemporal transcriptome landscape of planarian regeneration. They identified a novel pluripotent neoblast subtype and new spatially specific genes essential to tissue regeneration.

## Introduction

Understanding the fundamentals of tissue and organ regeneration is conducive to promoting the revolution of modern human medicine. The limited regenerative ability of the mammalian system has led researchers to resort to a kind of flatworm called planarian, with the capability of regenerating any missing part upon injury in a highly-organized manner. Thus, the planarian becomes an ideal model to study the in-depth mechanisms of regeneration. Its powerful regenerative ability originally benefits from a proliferative cell population containing pluripotent stem cells, also known as neoblasts, which are widely distributed in the whole body, accounting for 25–30% of the whole-worm somatic cells^1^. They can differentiate into nearly 40 types of cells, including nerve cells and germline cells, and are responsible for physiological homeostasis, growth and regeneration. Besides neoblasts, other cell types are also essential for the successful outcome of regeneration^2^. Thus, intricate coordination among many different cell types is required for the regeneration process.

Several important genes involving multiple signaling pathways have been shown to precisely regulate the regeneration process of planarians, enabling the planarian to accurately regenerate appropriate tissues after injury^3–5^. The establishment of polarity influences the regeneration of planarians, among which, for example, the expression of polarity genes regulates the establishment of anterior-posterior polarity (A-P)^6^. A long-lasting effort has been made to find the key regulatory factors essential to planarian regeneration. Based on the approaches being taken, most studies in the past can be divided into two classes, which are either knocking down the components of classic signaling pathways that have already been illustrated in other model systems, such as the *Wnt* signaling pathway^7,8^, or targeted screening efforts^9,10^. These methods were mostly focused on the genes with known functions in the planarian system, and the throughput of screening for genes with novel functions for planarian regeneration was very limited.

The single-cell RNA sequencing (scRNA-seq) technologies have facilitated the profiling of the transcriptomic cell atlas of regenerating planarians. Many cell types and essential regulatory factors related to regeneration have been uncovered based on the cell atlas^11–13^. However, for determining the spatial distribution of gene expression during the regenerative process, previous studies mainly employed the in situ hybridization based staining approaches, which are laborious and low-throughput. A high-throughput survey of the spatiotemporal landscape of both the gene expression patterns and intricate cellular coordination in the regeneration process is still lacking^14,15^. The recent advance in spatial transcriptomics (ST) technology can quantitatively delineate the spatial map of both polyadenylated transcripts and cells in a tissue section using barcoded oligo-dT arrays and standard histological bright-field imaging. Undoubtedly, it will be helpful to understand the dynamical changes in both spatial heterogeneity of cell distribution and cell-cell communication, and the characteristics of gene expression during planarian regeneration.

In this study, we established a comprehensive spatiotemporal landscape of the characteristic cell distribution and gene expression patterns of planarian regeneration (six time points), and the single-cell transcriptomic atlas of planarian regeneration (six time points plus an additional two). We generated three-dimensional (3D) transcriptomic maps via the integration of both ST and scRNA-seq data. We further unveiled an omnipotent stem cell cluster and identified multiple gene expression patterns associated with the regeneration of planarians. Using the spatial modules as a screening tool, polar genes with expression specific to wounds or the anterior/posterior regions of the planarian were identified to be essential to the regeneration. Importantly, spatial modules reveal a critical regulatory gene, *plk1*, for blastema formation and regeneration. Functional analysis demonstrated *plk1* as a key early response element for the initiation of blastema formation and essential for the success of subsequent regeneration events.

## Results

### The four-dimensional spatiotemporal transcriptomic atlas of the planarian *Schmidtea mediterranea*

**Fig. 1:**
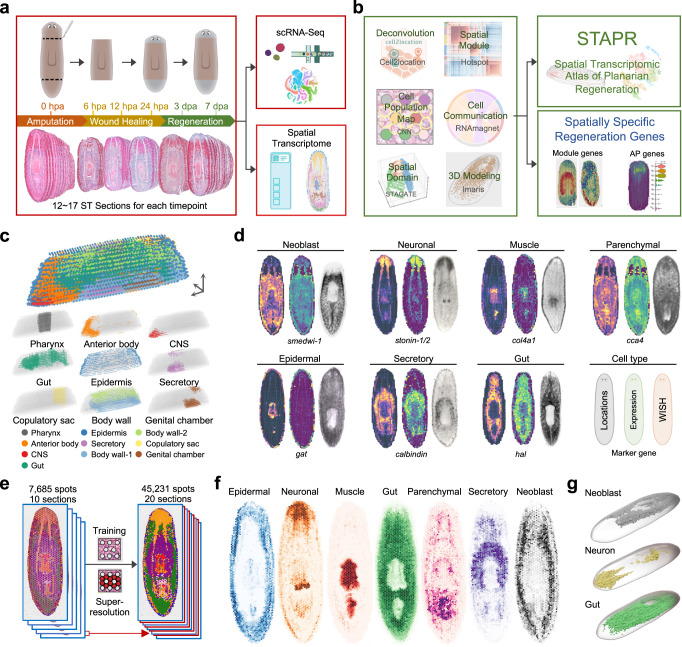
The four-dimension spatiotemporal transcriptomic cell atlas of planarian *Schmidtea mediterranea*. **a** Overview of the study design for multi-omics atlas of planarian regeneration. **b** Workflow depicting processing and content of analysis. **c** ST spots clustering using STAGATE according to spatial expression similarity and named by distribution characteristics. **d** Overlay of hematoxylin and eosin (H&E) staining of tissue sections and ST spots of each major cell type predicted by cell2location, in comparison to whole-mount in situ hybridization (WISH) staining of each cell marker. Scale bar, 500 µm. **e** High-resolution ST spots imputed by the deep learning procedure-based cell annotation, overlaying with H&E staining, and stacked to form a three-dimensional atlas. **f** Spatial distribution of major cell types with tiled spots from 20 consecutive sections. **g** Planarian virtual model illustrating major cell types reconstructed by Imaris. See also Supplementary Figs. 1–4.

To recapitulate the in-space molecular dynamics of the planarian regeneration process, we generated the spatiotemporal transcriptomic atlas of planarians across six time points of the regeneration process using 10x Visium technology. The samples include 17 whole-worm coronal cryosections at 0 h post-amputation (hpa), 17 at 6 hpa, 12 at 12 hpa, 12 at 24 hpa, 15 at 3 days post-amputation (dpa), and 16 at 7 dpa (Fig. 1a). Meanwhile, we generated a set of single-cell transcriptomic data from the same batch of samples collected from the identical time points as ST plus 2 and 5 dpa time points (Fig. 1a). In total, we collected transcription profiles of 59,506 spatial spots and 55,014 single cells from the regeneration process, obtaining a median of 21,388 UMIs/spot and a median of 3991 UMIs/cell (Fig. 1a, Supplementary Fig. 1a, b, and Supplementary Data 6). To reconstruct 3D cell distribution profiles of planarians at each regenerating time point, we took advantage of the latest data analysis tools such as Cell2location^16^, Hotspot^17^, STAGATE^18^, and Imaris (Fig. 1b). All our data are available as an online resource that can be used to screen for spatially specific genes related to regeneration (Fig. 1b) (STAPR, http://starp.space). In short, we established a comprehensive spatiotemporal transcriptomic cell atlas. This provides a virtual platform for instant visualization of genome-wide gene expression, spatially variable genes, and cell type distribution. Furthermore, it can also be used for studying the 3D tissue structures across the whole regeneration process of the planarian.

We constructed the 3D spatial substructures of spatial tissue represented by the ST at 0 hpa by an unsupervised tool STAGATE and projected the clusters into a 3D model. The results demonstrated that the spatial clusters were well consistent with the real spatial location of major tissues of the planarian shown by H&E staining (Fig. 1c and Supplementary Fig. 1c), indicating the effectiveness of the profiled atlas. Interestingly, the spatial distribution of the secretory system is segregated into upper and lower parts concerning the pharynx, and body wall muscle is also divided into two parts distributed in different spatial locations, with one part located close to the dorsal side and one to the ventral side (Fig. 1c). These results can be further used as the basis for exploring the dynamics of 3D tissue structures during regeneration.

We further estimated the fine-grained cell types in the spatial cell map of the planarian from spatial transcriptomic spots of each cryosection. Cell markers are important for identifying and classifying cell types. We systematically compared the cell type markers we identified based on both single-cell data and corresponding ST patterns with the established ones reported previously^11,12^. We were able to identify cell type markers that better characterize the six major cell types in terms of distinguishing cell clusters in Uniform Manifold Approximation and Projection (UMAP) results and defining tissue structure in ST results (Fig. 1d and Supplementary Fig. 1e). These cell markers include neuronal marker *stoning-1/2*, muscle marker collagen alpha-1(IV) chain (*col4a1*), parenchymal marker chloride channel accessory 4 (*cca4*), epidermal marker mitochondrial glycine amidinotransferase (*gat*), secretory marker *calbindin* and gut marker histidine ammonia-lyase (*hal*) (Supplementary Data 5). The cell types distribution matched well with the whole-mount in situ hybridization (WISH) staining of cell markers and Hematoxylin and Eosin (H&E) staining (Fig. 1d), suggesting the current spatial transcriptomic atlas can well capture the distribution of cell types. We also investigated the 3D pattern of cell type distribution, and found that their spatial distributions were continuous across sections and highly consistent with the expression of marker genes (Supplementary Fig. 2a, b). Moreover, at 6 hpa time point, the distribution of major cell types also showed similar spatial patterns as their marker genes (Supplementary Fig. 1f). And as expected, we found that, for example, neuronal and intestine cell types are well-separated, and barely any non-neuronal or non-intestine cell types in the brain or intestine.

To increase the cell-type annotation resolution, we utilized a deep-learning model of spatial transcriptomics data. Specifically, it aims to model the relationship between the annotated cell-type composition and the H&E staining images of each spot in the serial cryosections that have been sequenced. Based on that, we used the model to predict the cell-type composition of un-sequenced spots between the sequenced spots based on their corresponding H&E images, or the serial cryosections between the ones used for ST. At last, we were able to generate five times more new spots with cell-type annotation in the whole-worm ST data from 7685 of ten sections to 45,231 of 20 sections (Fig. 1e and Supplementary Fig. 1d). During the training stage, the accuracy of the pretraining model achieved 82.85% through the fivefold cross-validation (Supplementary Fig. 3) and the accuracy of a section-training model achieved nearly 90% after transfer learning (Supplementary Data 8). All the cryosections with enhanced cell-type annotation for a whole worm were aligned and stacked based on the characteristic pattern of tissue H&E staining (Fig. 1f). The resulting whole-worm spots representing seven major cell types showed increased resolution in comparison to the original ST data and were close to the real distribution of cell types in space (Fig. 1f). Using these enhanced spatial cell-type annotations, we further reconstructed a 3D high-resolution digital planarian model using Imaris software to demonstrate the 3D spatiotemporal distribution of each cell type, which could help to better reveal the dynamic changes of cell composition during regeneration (Fig. 1g and Supplementary Fig. 1d).

### Spatiotemporal dynamic cell populations during planarian regeneration

**Fig. 2:**
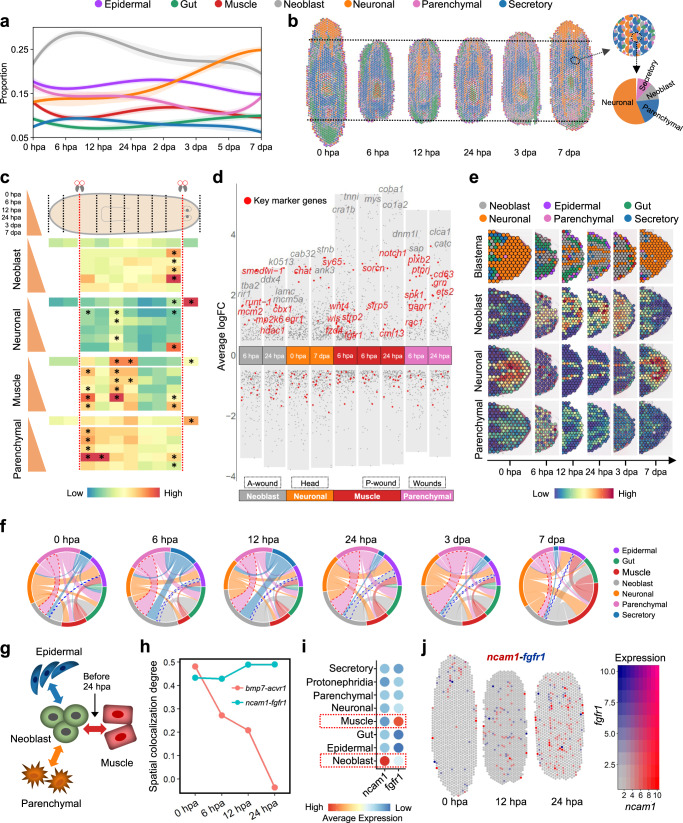
Spatial dynamics of cell types during regeneration. **a** Dynamic change of each cell population over the scRNA-seq of whole-body regeneration time courses. Dynamic change of each cell population over the scRNA-seq of whole-body regeneration time courses. We performed five times of 20% downsampling, and the standard deviation was shown as the error bands. Source data are provided as a Source Data file. **b** Spatial change of major cell types during the regeneration time courses. ST spot is illustrated as a pie chart of major cell types. **c** Average prediction scores and spatial enrichment test of major cell types along the anterior-posterior axis of planarian during the regeneration. Hypergeometric test (one-sided), *p* value <0.05*. Source data are provided as a Source Data file. **d** Volcano plot showing spatial differential genes that are up- and down-regulated in neoblast, neuronal, muscle, and parenchymal clusters in wound region at the A-wound and P-wound, anterior wound and posterior wound. Key regulatory genes are indicated in red, while other genes are indicated in gray. Source data are provided as a Source Data file. **e** Tissue sections of the anterior wound area showing the cell type with the maximum prediction score of ST spots, and prediction scores of neoblast, neuronal, and parenchymal of ST spots. **f** Chord diagram showing predicted signal correlation of pairwise cell types in ST spots in all regeneration time points. **g** Schematic representation showing the communication between neoblast and other cell types in planarian. **h** Spatial colocalization degree of ligand-receptor pairs *bmp7-acvr1* and *ncam1-fgfr1* before 24 hpa. Source data are provided as a Source Data file. **i** Dotplot of ligand-receptor pair NCAM1-FGFR1 showing the average expression in major cell types. **j** Co-expression pattern of ligand-receptor pair *ncam1-fgfr1* at 0, 12, and 24 hpa respectively. See also Supplementary Fig. 5.

The spatiotemporal transcriptomic atlas has the power in dissecting the dynamics of cellular changes during regeneration. Our results showed that several cell populations during planarian regeneration underwent dynamic and asynchronous changes. The neoblast population initiated a sharp increase at 6 hpa, reached a maximum at 12 hpa in the early stage of the regeneration process, and then gradually decreased (Fig. 2a). At a later stage, there was a gradual increase of neuronal cells after 24 hpa and a sharp increase of parenchymal cells at 7 dpa (Fig. 2a). ST data also showed an increase of neuronal cells in the anterior region from 12 hpa and an increase of parenchymal cells in the trunk region (Fig. 2b). These results indicated that the early wave of neoblast proliferation is essential for the regeneration, and growth of newly differentiated cell types mostly happened at the later stages at different spatial locations.

In order to quantify the regional change of cell composition during the regeneration, we divided the whole worm into ten sections along the anterior-posterior axis of the planarian, and average prediction scores and spatial enrichment tests of major cell types were applied in each section of the planarian during the regeneration (Fig. 2c). Neoblasts were increased at both anterior and posterior facing wounds during the regeneration until 7 dpa. In particular, a significant enrichment at the anterior wound site was observed at a very early time point (6 hpa) and maintained at a higher level till 7 dpa. In contrast, the neoblast number at the posterior wound site was relatively low from 6 to 24 hpa but had an increase post 3 dpa even though not reaching statistical significance (heatmap in Fig. 2c and Supplementary Data 7). In particular, a significant number of neoblast-related genes were activated at the anterior wound site compared to the posterior one (asterisk symbols in Fig. 2c). These results indicate that the neoblasts are involved in the regeneration process of both head and tail, whereas the head is regenerated priorly over the tail. Neurons were enriched in the head region at 7 dpa (Fig. 2c and Supplementary Fig. 5a), which indicates that the neuronal structure of the planarian has regenerated at 7 dpa, and neoblasts play an important role during this process.

The marker genes of major cell types include many previously reported important ones related to planarian regeneration. By overlapping the spatial differential genes at the anterior and posterior wound regions with the marker genes of each major cell type, we found a range of genes including some key regulatory genes being upregulated specifically at the wound regions during the regeneration, such as the neoblast marker genes of *smedwi-1* and *hdac1*^19,20^ at the anterior region, neuronal marker genes of *chat* and *sy65* related to neurodevelopment at the head region, muscle marker genes of *scorn, cml13*, and *sfrp5*, planarian positional information related genes of *fzd4*, *wnt4*, and *wls*^21^, and parenchymal marker genes of *plxb2*, *grn*, and *spk1*, etc. (Fig. 2d). Also, some marker genes are specifically distributed at the anterior or posterior wound site (Fig. 2d). These include some of the reported genes essential for regeneration, such as *hdac1*^19,20^, *runt-1*^10,22^, *myoD*^23^, and *wls*^21^, suggesting the power of this dataset to screen for regeneration-related genes. Cell composition of blastema during the regeneration confirmed that neoblasts and parenchymal cells increased their presence at the anterior site and contributes to the regeneration of the head (Fig. 2e and Supplementary Fig. 5b–d, f).

To explore the spatial organization of all tissues during the regeneration, the pairwise cell-type prediction signal correlation was calculated to highlight the significant co-occurrence of cell types in each ST spot. The neoblasts and neuronal cells occurred in close vicinity in the early regenerating stages (Fig. 2e, f), suggesting the differentiation of neoblasts to nerve cells to regenerate the head. Through the analysis of the communication network (Supplementary Fig. 5e), we found that neoblasts show communication with epidermal and parenchymal cells throughout planarian regeneration. Specifically, neoblasts prefer to communicate with muscle cells during the early stage of generation (Fig. 2g and Supplementary Fig. 5e), which was consistent with the results of a previous study^23^. We identified two ligand-receptor pairs, *bmp7-acvr1*, and *ncam1-fgfr1*, which had significant communication from muscle cells to neoblasts. These ligand-receptor pairs are associated with cell proliferation, differentiation, migration, and other biological processes^24,25^. By applying the spatial colocalization test, we found that *ncam1-fgfr1* had an increased trend of colocalization before 24 hpa (Fig. 2h). We speculate that *ncam1-fgfr1* may be involved in the communication between neoblasts and muscle cells from 6 hpa to 24 hpa. Moreover, *ncam1-fgfr1* is highly expressed in neoblasts or muscle cells (Fig. 2i), suggesting that *ncam1-fgfr1* signaling may regulate the delivery of the signaling from muscle cells to neoblasts for regeneration-associated differentiation. *fgfr1* was shown to be implicated in the signaling system controlling stem cell differentiation and further planarian regeneration^26^. Furthermore, *NCAM/FGF*-receptor interaction is critical for cell proliferation and migration during zebrafish lateral line phylogeny^27^. Since the co-expression of *ncam1* and *fgfr1* has a tendency to increase near the anterior region from 12 to 24 hpa (Fig. 2j), it is speculated that *ncam1-fgfr1* may be involved in the head regeneration of planarian. The spatial dynamic changes of other tissue types can also be visualized through 3D reconstructed model (Supplementary Fig. 5g).

### The atlas defines a neoblast subpopulation with a stronger differentiation potential

**Fig. 3:**
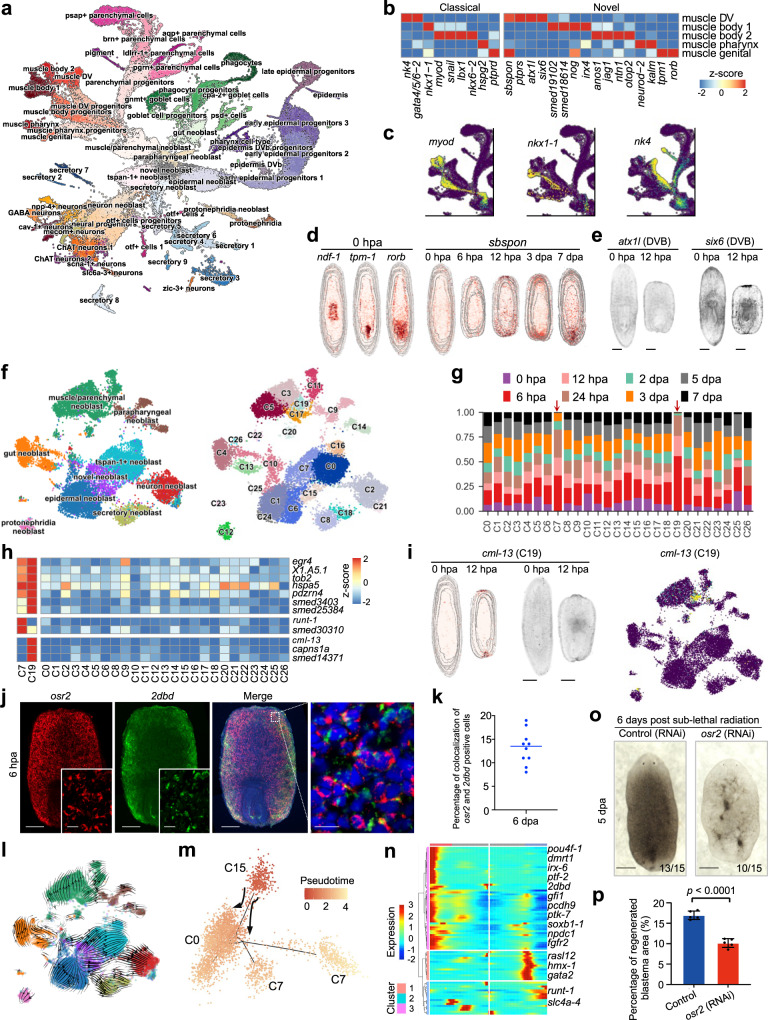
A neoblast sub-cluster identified as the most potent stem cell for planarian regeneration. **a** UMAP demonstrates sub-cell types. **b** The classical and identified markers for muscle sub-cell types. **c** The expression pattern of classical marker genes for muscle sub-cell types. **d** The expression pattern of *ndf-1*, *tpm-1*, and *rorb* at 0 hpa ST data (left) and *sbspon* during regeneration (right). **e** The WISH staining of muscle DV markers, *atx1l*, and *six6*, at 0 and 12 hpa. Scale bar, 500 μm. **f** UMAP demonstrates neoblast subpopulations (left) and neoblast sub-cluster (right). **g** Cell proportions of neoblast subpopulations during regeneration. Source data are provided as a Source Data file. **h** The identification of marker genes for C7 and C19. **i** The expression of C19 marker gene*, cml-13*, in 0 and 12 hpa ST data (left). The WISH staining of *cml-13* at 0 and 12 hpa (middle). Scale bar, 500 μm. The expression of *cml-13* in scRNA-seq data (right). **j** Fluorescent in situ hybridization (FISH) showing expressions of C15 markers, *osr2*, and *2dbd*, in 6 hpa samples. Scale bar, 300 and 20 µm for the enlarged field. **k** The percentage of colocalization of *osr2* and *2dbd* positive cells. Data were mean ± S.D. and *n* = 10 animals. Source data are provided as a Source Data file. **l** Velocity force field showing the average differentiation trajectories (velocity) for cells located in different parts of the UMAP plot. **m** Pseudo-time of neoblast cells inferred by Monocle2. **n** Three clusters of branch-dependent genes identified by BEAM. **o** Bright-field image showing control and *osr2* RNAi planarians at 5 dpa, with 6 days post sublethal radiation. Scale bar, 500 μm. Bottom left number, planarians with the phenotype of total tested. **p** The percentage of regenerated blastema area to the whole body of the control and *osr2* RNAi planarians. Error bars represent standard deviation. Data were mean ± S.D. and *n* = 5 animals in each group. The *p* values were determined using a two-sided unpaired Student’s *t*-test (right). Source data are provided as a Source Data file. See also Supplementary Figs. 6, 7.

We next used our large-scale single-cell transcriptomic atlas to systematically study the cellular changes during regeneration. A total of 55,014 single cells were clustered into seven major cell types consisting of neoblast, neuron, muscle, gut, secretory, epidermal, and parenchymal cells (Supplementary Fig. 6a, Supplementary Data 1, and Supplementary Data 6), representing the main cellular components of the planarian. The marker genes for the subclusters of each major cell type were elucidated and compared with those previously reported in other studies (Fig. 3a and Supplementary Fig. 6b, c)^12^. RNA velocity analysis of all cell types showed that the neoblast was the root of the trajectory and UMAP revealed that progenitor cell clusters were located between differentiated cells and neoblast clusters (Supplementary Fig. 6d). Moreover, we identified eight subtypes of muscle cells (Fig. 3a) and several markers (Fig. 3b, c). Interestingly, based on the marker gene expression in spatial transcriptome data, we showed the specific spatial distribution of muscle cells. For example, *ndf-1*, the marker gene of muscle pharynx, was mainly expressed in the pharynx area, and the marker genes of muscle genital, *tpm-1* and *rorb*, were mainly in the genital area (Fig. 3d). We also identified *sbspon*, which was mainly expressed in muscle DV and muscle genital, with an enriched expression at the wound area from 12 hpa to 7 dpa (Fig. 3d). This result indicates that the muscle subclusters represented by *sbspon* may have an important role in wound healing. Moreover, the markers of muscle DV (*atx1l* and *six6*) showed major expression in the wound area (Fig. 3e), suggesting important regulatory roles of muscle DV in the wound healing during regeneration process. Knockdown of one of these marker genes, NK homeobox 4 (*nk4*), resulted in the loss of midline symmetry and fusion of eye spot phenotype (Supplementary Fig. 6e), which is consistent with a previous study^28^. These findings suggest that muscle DV is critical in re-establishing the midline symmetry during planarian regeneration.

Moreover, one study^29^ recently identified a transient muscle cell type with high expression of the notum gene during regeneration. We integrated the muscle cells from our scRNA-seq data with the muscle cells profiled by Benham-Pyle et al. and found a cluster that showed high expression of notum and similar spatiotemporal distributions with the transient muscle cell type (Supplementary Fig. 7a–i).

We further classified the key and highly heterogeneous neoblast population. Through an unsupervised clustering analysis, apart from nine known subcellular types (such as epidermal, neuronal, and muscle neoblasts), 26 heterogeneous subclusters were identified (Fig. 3f). One of these sub-cluster, cluster 7, increased in its population rapidly at 6 hpa and maintained until 24 hpa (Fig. 3g, h). The cells in this cluster are marked by the expression of *runt-1* gene, which has been reported as one of the wound healing genes and can promote heterogeneity in neoblasts near wounds^10,22^. Furthermore, the cells in cluster 19 increased rapidly at 6 hpa and decreased gradually from 12 hpa to 7 dpa (Fig. 3g, h). Staining results further confirmed the enrichment of C19 in the wound area during regeneration, suggesting that C19 might participate in early wound response (Fig. 3i). These results imply that clusters 7 and 19 might participate in wound response and tissue regeneration.

Interestingly, we also found a neoblast subpopulation (classified as C15 and named *osr2*^*+*^ neoblast) with specific marker genes of *odd-skipped-related 2* (*osr2)* and *nuclear receptor 2DBD* (*2dbd)* (Fig. 3j, k and Supplementary Data 2). The mRNA velocity analysis on neoblasts supports the differentiation trajectory from the *osr2*^*+*^ neoblast to the Nb2 subpopulation, a previously identified subtype of neoblast capable of increasing the viability of lethally irradiated planarian^13^ (Fig. 3l). The same predicted trajectories were acquired by Monocle (Fig. 3m). The trajectory pattern indicates that the *osr2*^*+*^ neoblast has stronger differentiation potential than other neoblasts. We also identified some significant branch-dependent genes, such as *pou4f-1* and *soxb1-1*, which might influence the differentiation of the *osr2*^*+*^ neoblast (Fig. 3n). To investigate the role of *osr2*^*+*^ neoblast in regeneration, we used a sublethal dose of radiation to irradiate the regenerated planarian and observed that the planarians with *osr2* depletion regenerated much slower than the control (Fig. 3o). Statistical analysis showed that the regenerated new tissue was significantly less in *osr2* knockdown worm than in control (Fig. 3p). In addition, *osr2* knockdown did not affect the survival of neoblasts during tissue homeostasis (Supplementary Fig. 6f, g). This result suggests an essential role of *osr2*^+^ neoblast in the planarian regeneration process.

Thus, through analyzing the heterogeneity of planarian cell types, we identified several subclusters of major cell types, in particular the subgroups with pluripotent potential, that might affect the regeneration of planarians.

### Spatial modules identify essential regulatory genes for regeneration

**Fig. 4:**
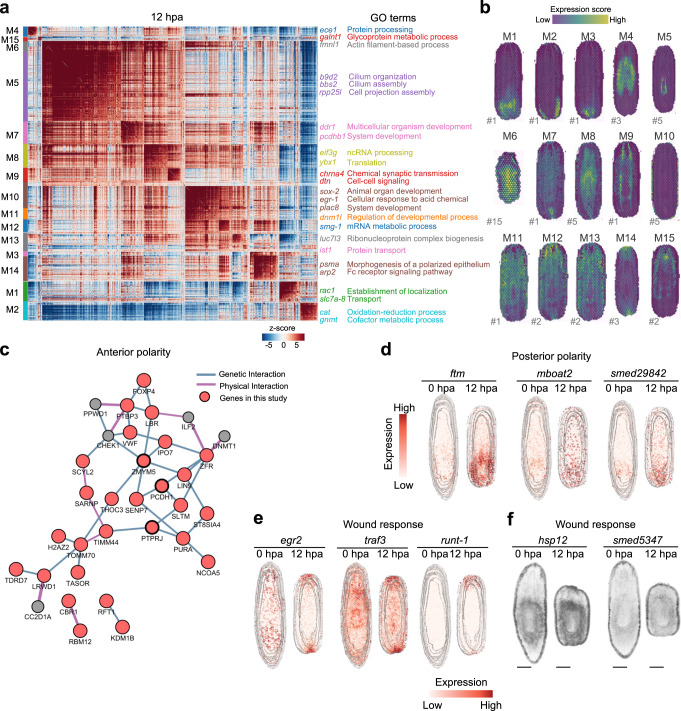
Spatial module can help to screen for regeneration-related genes. **a** Correlation heatmap of functional gene modules identified by Hotspot analysis in 12 hpa sample. Each row and each column represent a module marker gene, and *Z*-score indicates the correlation between module marker genes. Right panel: GO analysis showing the top function terms of each module. **b** The expression pattern of each module identified in (**a**). The “#number” next to the sections represents the number of each section. **c** The gene-regulated network of anterior polarity gene modules. **d** The expression levels of posterior polarity genes, *ftm, mboat2*, *and smed29842*, in 0 and 12 hpa ST data. **e** Spatial distribution of wound-induced genes *egr2*, *traf3*, and *runt-1* at 0 and 12 hpa. **f** WISH staining of polar genes, *hsp12* and *smed5347*, identified through the polarity gene module. Scale bar, 500 μm. See also Supplementary Fig. 8.

To investigate whether gene expression has different spatial characteristics during regeneration, we clustered the high variable genes of spatial transcriptome data and obtained multiple gene expression modules with different functions at each time point (Fig. 4a and Supplementary Fig. 8a–h). From 12 hpa spatial transcriptome data, we detected 15 gene expression modules (Fig. 4a and Supplementary Data 3), and through Gene Ontology (GO) function enrichment analysis, we found several gene modules with potential regulatory roles in regeneration (Fig. 4a). For example, the genes in module 7 were enriched in multicellular organism and system development, and the genes in module 9 were enriched in cell-cell signaling and related pathways, indicating that different gene modules may play differential regulatory roles in regeneration, in particular for the genes with spatial specific expression in some tissues. For instance, the expression of certain A-P polarity genes regulates the establishment of planarian polarity during regeneration^5,30,31^. We used the genes of each module to score each ST spot to determine the expression pattern and identified three gene modules with a distinct pattern of polar expression (Fig. 4b). Among them, module 15-related genes were mainly expressed in the anterior area of the planarian, with functional enrichment in the glycoprotein metabolic process, while the genes with posterior polarity were enriched in protein transport and related functions. Moreover, the genes mainly expressed in the wound area were enriched in the morphogenesis of a polarized epithelium, indicating the important function of these genes during the regeneration. To further determine the key regulators for regeneration, an interaction network was constructed to decipher the regulatory relationships, and the potentially critical hub genes were detected, including *pcdh1, ptprj, and zmym5* (Fig. 4c). Meanwhile, the ST data were further compared with that of 0 hpa in aims to detect the important polar genes in regulating regeneration. Combining with the interaction network and differential expression analysis, we found several genes with significant expression changes during regeneration. For example, compared with 0 hpa ST data, the expression of *mboat2*, *ftm*, and *smed29842* was elevated and mainly enriched in the posterior area at 12 hpa (Fig. 4d). Furthermore, several genes were identified to be mainly expressed in the wound area, including *egr2*, *traf3*, *runt-1, hsp12*, and *smed5347*, even though they showed expression throughout the whole body at 0 hpa (Fig. 4e, f and Supplementary Data 4). These results suggest that the atlas help to visualize and understand the dynamic changes of gene expression, which makes it feasible to identify the key regulatory genes important for the regeneration of different tissue parts of the planarian.

### *plk1* gene is essential in planarian regeneration

**Fig. 5:**
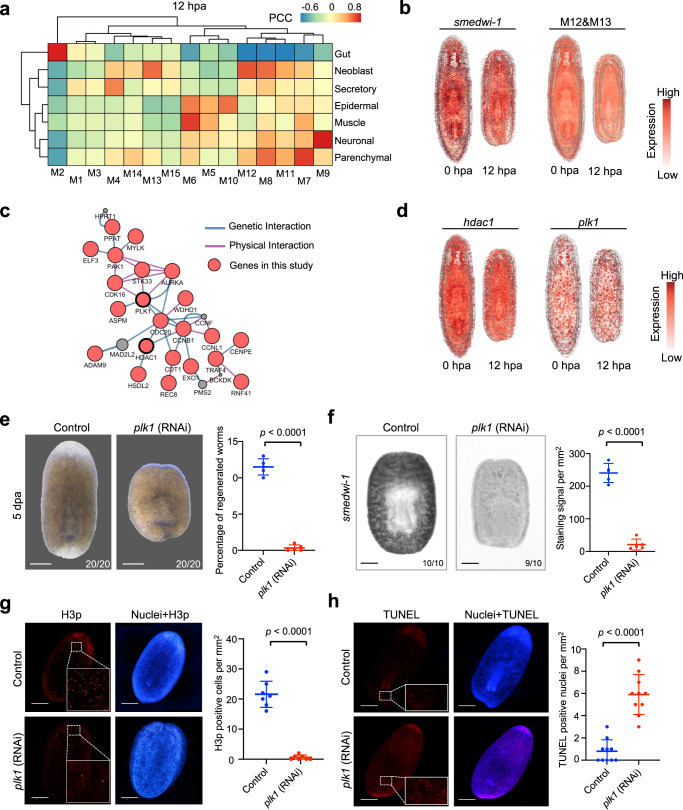
Spatial modules identify key regulatory genes that are essential for regeneration. **a** Heatmap showing the correlation between modules (Fig. 4a) and cell types (predicted by cell2location). Source data are provided as a Source Data file. **b** Spatial distribution of *smedwi-1* and neoblast-related modules genes expression score. **c** Gene network of neoblast-related module reveals hub genes. **d** Spatial expression of key regulatory genes *hdac1* and *plk1* in 0 and 12 hpa ST data. **e** Bright-field image showing control and *plk1* knockdown planarians at 5 dpa. Scale bar, 500 μm. Bottom right number, planarians with the phenotype of total tested. Error bars represent standard deviation. Data were mean ± S.D. and *n* = 5 animals in each group. The *p* values were determined using a two-sided unpaired Student’s *t*-test (right). Source data are provided as a Source Data file. **f** WISH staining of *smedwi-1* in control and *plk1* knockdown planarians at 3 dpa. Scatter plot of *smedwi-1* positive cells in each group. Scale bar, 300 µm. Bottom right number, planarians with the staining pattern of total tested. Error bars represent standard deviation. Data were mean ± S.D. and *n* = 5 animals in each group. The *p* values were determined using a two-sided unpaired Student’s *t*-test (right). Source data are provided as a Source Data file. **g** Immunofluorescence of H3p in control and *plk1* knockdown planarians at 5 dpa. Scale bar, 500 μm (left). Statistical analysis for H3p immunostaining. Error bars represent standard deviation. Data were mean ± SD and *n* = 7 animals in each group. The *p* values were determined using a two-sided unpaired Student’s *t*-test (right). Source data are provided as a Source Data file. **h** Whole-mount TUNEL assay of control and *plk1* knockdown planarians at 5 dpa. Scale bar, 500 µm (left). Quantification of TUNEL^+^ nuclei/mm^2^ at 5 dpa. Error bars represent standard deviation. Data were mean ± SD and *n* = 10 animals in each group. The *p* values were determined using a two-sided unpaired Student’s *t*-test (right). Source data are provided as a Source Data file. See also Supplementary Fig. 9.

As neoblasts play an essential role in the regenerative ability of planarians^13,32^, we then analyzed the gene modules with spatial distribution similar to that of neoblasts. The average expression of modules 12 and 13 was found to have a distinctly high correlation with that of neoblasts (Fig. 5a, b). To further explore the key genes in regeneration, we constructed the interaction network with genes of modules 12 and 13. Several hub genes, including *hdac1* and *plk1*, with spatial distribution similar to neoblasts and *smedwi-1* (Fig. 5b–d and Supplementary Fig. 9a), were identified. Previous reports had demonstrated that *hdac1* depletion led to an inability to launch a regenerative response after amputation^33^, and *plk1* is an evolutionary conserved Ser kinase and regulates the cell cycle process in human^34^. Thus, these several hub genes are the potential regulators of the neoblast and further regeneration process.

To determine whether or not *plk1* influences the regeneration process, we performed *plk1* knockdown and the result showed that the regeneration was completely failed and almost no new tissue was regenerated in *plk1*-depleted planarians (Fig. 5e), indicating an essential role of *plk1* in the regeneration process. Moreover, we found that most *smedwi-1*^+^ cells disappeared at 3 dpa upon *plk1* knockdown (Fig. 5f). The cell proliferation was apparently affected as the staining density of phosphorylated histone 3 (H3p) was significantly decreased in *plk1* RNAi planarian (Fig. 5g). These results suggested that *plk1* affects the regeneration process through regulating the normal function of neoblasts, such as proliferation. We further revealed that cell death determined by a TUNEL assay was significantly increased upon *plk1* knockdown (Fig. 5h), supporting the essential roles of *plk1* in planarian regeneration. The enhanced cell apoptosis, together with the decreased cell proliferation upon *plk1* depletion, led to neoblast exhaustion and consequent failure of regeneration. In addition, we also identified several planarian-specific genes without annotation, such as *smed30000353* (*nb0353*) and *smed30003006* (*nb3006*) (Supplementary Fig. 9a, b). Similar to the *plk1*-depleted phenotype, *nb3006* knockdown resulted in neoblast exhaustion as shown by WISH staining of *smedwi-1* and IF staining of H3p (Supplementary Fig. 9c–e), as well as regeneration failure phenotype, suggesting that it is also critical in the planarian regeneration. These results demonstrate the powerfulness of our ST data resource in identifying both conserved and planarian-specific genes critical for planarian regeneration, which provide the potential candidates for tracing their higher mammal homologs.

### scRNA-seq data uncover the effects of *plk1* depletion on the generation of stem cells of planarian

**Fig. 6:**
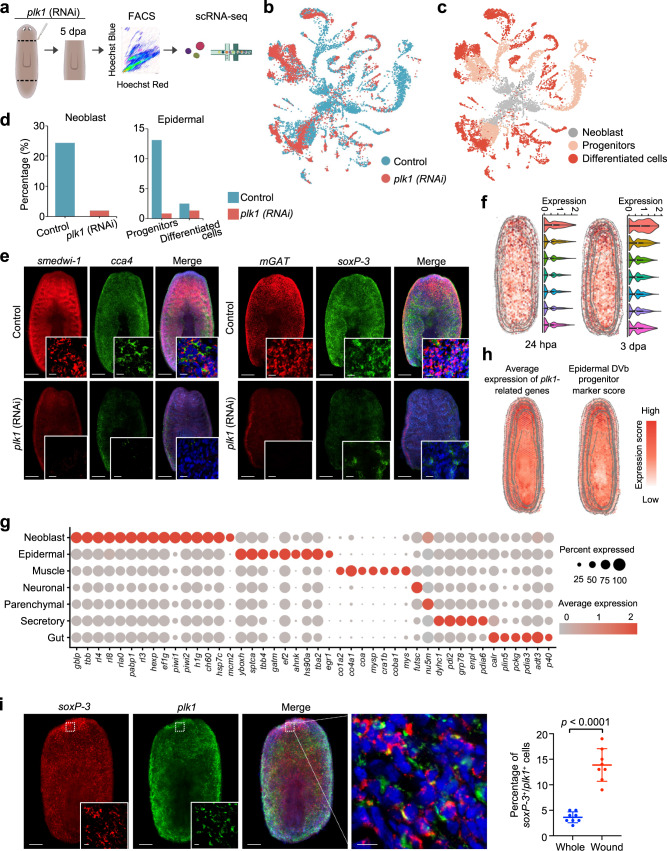
*plk1* is essential for planarian regeneration. **a** Overview of study design for *plk1* (RNAi) single-cell atlas of the planarian. **b** UMAP visualization of control and *plk1* knockdown scRNA-seq samples at 5 dpa. **c** The location of neoblasts, progenitor, and differentiated cell clusters was shown on the UAMP plot. **d** Percentage of cells allocated to different cell types in control and *plk1* knockdown planarians at 5 dpa. Source data are provided as a Source Data file. **e** FISH showing the expressions of *smedwi-1*, *cca4, mGAT*, and *soxP-3* in the control and *plk1* knockdown planarian at 5 dpa. Scale bar, 300 μm and 20 µm in the enlarged field. **f** The expression pattern of *plk1* at 24 hpa and 3 dpa during regeneration. *n* (24 hpa from top to bottom) = 463, 1426, 1885, 1999, 1940, 1719, 481; *n* (3 dpa from top to bottom) = 373, 1084, 1418, 1867, 2069, 1671, 740. The middle lines of the boxes represent the medians of datasets (50th percentile). The upper and bottom lines of the boxes are, respectively, the upper quantile (25th percentile) and the lower quantile (75th percentile) of the data. The whiskers mark the upper and lower limits of these datasets. **g** Dotplot showing the expression of differentially expressed genes and the expression of genes with expression patterns similar to *plk1*, in different cell types. **h** The gene expression score and cell type distribution at 3 dpa. The genes with a expression pattern similar to *plk1* was detected by Hotspot, and the cell type distribution were predicted by markers. **i** FISH showing co-expressions of *soxP-3 and plk1* at 5 dpa. Scale bar, 200 μm and 20 µm in the enlarged field. Quantification of the percentage of *soxP-3*^+^ / *plk1*^+^ cells mm^2^ of whole tissue fragment and near-wound area. Error bars represent standard deviation. Data were the mean ± SD. and *n* = 8 animals in each group. The *p* values were determined using a two-sided unpaired Student’s *t*-test (right). Source data are provided as a Source Data file. See also Supplementary Fig. 10.

To explore the underlying mechanism for *plk1* to regulate planarian regeneration, we performed single-cell transcriptome sequencing on *plk1*-depleted planarian samples at 5 dpa during regeneration (Fig. 6a). After initial quality control, we acquired single-cell transcriptomes of a total of 7941 cells from the *plk1* RNAi sample and characterized the cell types using the single cell data of control samples by Scanpy *Ingest* function (Supplementary Fig. 10a). Consistent with the phenomena of an increase in apoptosis, and decreases in both cell proliferation and *smedwi-1*^*+*^ cells (Fig. 5f–h), we observed that the cells with differentiation potential almost disappeared in the *plk1* RNAi sample. To reveal the effect of *plk1* knockdown on different cell types, we compared the composition of cell types between control and *plk1* RNAi samples. The results showed that upon *plk1* knockdown, the number of epidermal cells and neoblasts decreased the most, and also the number of the progenitors of epidermal, parenchymal cells, as verified by fluorescent in situ hybridization (FISH) staining using cell markers (Fig. 6b–e). These results suggest that *plk1* deficiency leads to the depletion of the stem cell pool in the planarian. We further analyzed the *plk1* spatial expression pattern at different time points during regeneration and found that *plk1* was highly expressed in the anterior area from 24 hpa to 3 dpa (Fig. 6f). Based on this observation, we speculated that *plk1* might be able to affect the function of other anterior-specific cell types in addition to neoblast. Through differentially expressed analysis on single-cell transcriptome data between control and *plk1* knockdown samples, we found that the *plk1*-affected genes were mainly expressed in neoblast and epidermal cells (Fig. 6g and Supplementary Fig. 10b, c). Moreover, *plk1* influenced some downstream genes in the gene interaction network or the ST gene modules, such as *mcm2*, *h1g* (histone H1), and *egr1* (Fig. 6g), which are essential for the development and regeneration processes^35^. We also used the marker genes to predict the spatial distribution of various cell subtypes and found that the expression pattern of *plk1*-affected genes was similar to that of the epidermal DVb progenitor cells (Fig. 6h and Supplementary Fig. 10d–f). Furthermore, *soxP-3* and *plk1* positive epidermal progenitor cells were indeed more enriched at the wound site (Fig. 6i), indicating the potential role of *plk1* in the epidermal progenitor at the wound region. All these results suggest that *plk1* might regulate the regeneration of planarian by regulating the proliferation of neoblast cells and the progenitor cells like epidermal progenitor cells.

Taken together, we identified *plk1* as a regulator for planarian regeneration through single-cell and spatial transcriptomics analyses, and demonstrated that *plk1* is critical to the function of neoblast as evidenced by the findings that *plk1* deletion could lead to exhaustion of neoblast and progenitor cells due to the reduced cell proliferation together with the increased apoptosis.

## Discussion

In this study, through profiling so far the largest pool of single cells in planarian, we established a comprehensive spatiotemporal transcriptomic atlas for describing the dynamic difference in both cell types and gene expression during regeneration. Based on this atlas, the 3D distribution of the major cell types, including neoblast, neuronal, parenchymal, epidermal, muscle, gut, and secretory cells, and specifically expressed genes at different regeneration stages were successfully defined. Moreover, subclusters of major cell types, such as muscle body and muscle genital, were identified. Our technical approaches and findings provide valuable platforms and resources for systematically delineating the regeneration process of planarians at the transcriptiomic level. To make this resource easily accessible, we have built an interactive website that allows users to query and interpret all the key data.

We identified a new pluripotent cell subgroup marked by *osr2*, and the knockdown of *osr2* in the irradiated planarian resulted in a delayed regeneration phenotype. Previous studies have shown that neoblast is a complex mixture of PSCs and lineage progenitors, and a single transplant of Nb2 neoblast cell can multiply into stem cell colonies and rescue stem cell-depleted planarians^13^. By clustering neoblast cells, we not only observed similar results, but also identified *osr2*^*+*^*/2dbd*^*+*^ pluripotent neoblast cluster that might affect regeneration. It has been known that *osr2* is a homolog of the *Drosophila* odd-skipped family, encoding zinc-finger transcription factors with widespread roles in embryonic development. Its orthologous gene in *Caenorhabditis elegans* is involved in gut development^36^, and mammal homologs (*osr1* and *osr2*) affect the development of the heart, urogenital, and secondary palate^36^. We found that the regeneration of *osr2* knockdown planarians was delayed after sublethal dose X-ray of irradiation, and the cell differentiation trajectory analysis indicated that the *osr2*^*+*^ neoblast can generate Nb2 and other neoblast cells^13^. Therefore, we propose a model in which the neoblast cells can generate the lineage branches to regulate planarian regeneration.

To comprehensively explore the key genes that affect the regeneration of planarians, we systematically analyzed the spatial- and cell-type-specific expression patterns. Several spatial gene expression modules related to wound regions or polarity (dorsal-ventral or anterior-posterior axis) were identified. In addition to several reported essential genes for regeneration, such as *runt-1*^10,22,37^ and *egr2*^38^, we further revealed other genes (Supplementary Data 3), including *smed29842* and *smed5347*, displaying similar spatial expression distribution to the above-reported ones. In addition, according to the spatial transcriptomics analysis, we also found several markers of muscle DV mainly expressed at the wound site, such as *nk4*. Similar to Scimone et al.^28^, we also found that *nk4* RNAi resulted in the loss of midline symmetry and fusion of eye spot phenotype. Besides that, we also found several markers of muscle DV, such as *atx1l* and *six6*, which are mainly expressed at the wound site. Although the knockdown of each of these genes did not affect wound closure, their synergistic effects on wound healing and regeneration deserve further investigation.

 One report showed that *plk1* participates in mouse muscle regeneration^39^. Our findings demonstrated a function of *plk1* in the homeostasis of the neoblast. Deficient *plk1* led to decreased neoblast and its derived progenitor cell populations during planarian regeneration. Although we found several genes with downregulation upon *plk1* knockdown and may be the potential downstream targets of *plk1*, the signaling pathways underlying *plk1* function, in particular how to affect the differentiation potential of neoblast, needs to be further explored. We meanwhile found genes with spatial expression patterns similar to that of *plk1*, including *nb0353* and *nb3006*, with essential roles in planarian regeneration. As these two genes do not exist in mammals and may be lost during the evolution process, it is worth of further study if re-expressing these genes in mammalian cells could affect their regeneration ability.

In our paper, we mainly focused on the seven major cell types in the spatial transcriptomic data, and have confirmed their spatial distributions by WISH staining of marker genes (Fig. 1d). We also employed cell2location to perform a more resolved deconvolution on the basis of 61 subtypes shown in Fig. 3a. As expected, we found that many inferred subtype spatial distributions were consistent with the corresponding expressing of previously reported gene markers during planarian regeneration (Supplementary Fig. 4). For example, we found that three neuronal subtypes (ChAT neurons 1, ChAT neurons 2, and GABA neurons) exhibited distinct spatial patterns (Supplementary Fig. 4a, b). Specifically, ChAT neuron 1 is mainly enriched in the brain and ventral nerve cord, ChAT neuron 2 is enriched around the pharynx region and GABA neurons are located at the head region. These spatial patterns have been confirmed by WISH staining of marker genes in previous studies^12^. Moreover, we also observed distinct spatial distribution of muscle subtypes (Supplementary Fig. 4c). More interestingly, the inferred subtypes could help us to study the spatial changes of these cell types during regeneration. For example, we found that the neoblasts were enriched in the wound region at 6 hpa and throughout the regeneration process (Supplementary Fig. 4d). However, many subtypes are difficult to be characterized by one or two genes, which makes it very difficult for us to verify the results experimentally. Moreover, several subtypes showed low cell abundance throughout the planarian body, which also makes deconvolution analysis difficult.

Overall, our work provides a valuable resource of planarian regeneration-associated 3D profiles of dynamic cellular components and gene expressions, key regulatory factors, and signaling pathways, which opens a window to clearly see the detailed regulation of planarian regeneration and has fundamental significance in both planarian and mammalian regeneration research.

## Methods

### Planarian culture and irradiation treatment

The asexual CIW4 strain and sexual S2F8b strain of *Schmidtea mediterranea* were maintained at 20 °C in Montjuïch salts as previously described in Ref. ^40^. Animals were fed weekly with homogenized pig liver. For all experiments, animals were starved for at least 1 week. The RS 2000 Small Animal Irradiator was used to expose animals to 1,250 rads for sublethal irradiations.

### Gene knockdown by RNAi feeding

Genes were cloned and amplified from a CIW4 whole-animal cDNA library. RNAi was performed by feeding using double-stranded RNA (dsRNA) synthetized by in vitro transcription as previously described in Ref. ^41^. In summary, genes were amplified with T7 sequences flanked at both ends to generate a dsRNA template, and in vitro transcription was performed using the T7 polymerase system (Promega, P2075). Planarians were fed by dsRNA mixed with pig liver paste at a ratio 1:4 for three times at 3-day intervals. Amputation was performed at head and tail regions (pre- and post-pharyngeal) 24 h after the last RNAi feeding. The *C. elegans unc-22* gene was used as a negative RNAi control. Primers used for the dsRNA synthesis are listed in Supplementary Data 5. ImageJ (V1.5) were used to measure the pixel area of regenerated blastema divided by that of the whole tissue fraction.

### Single-cell RNA-seq library construction

Single-cell suspension was prepared as previously described with some modifications^13^. Briefly, planarians were finely diced on ice plates and washed off the plate with CMFB buffer, followed by digestion with 1% trypsin in CMFB (CMF + 0.5%BSA) for 8 min at room temperature with gentle agitation, and then pipetting up and down the suspension to break up the clumps. After being strained through a 40 mm filter, cells were pelleted by centrifugation at 290×*g* for 5 min at 4 °C and stained with Hoechst 33342 (Thermo, 62249) for 30 min at room temperature. Before loading onto the MoFlo cell sorter, cells were strained again and then Propidium iodide (Thermo, J66584.AB) was added to discriminate dead cells. For single-cell RNA-seq from the 10x Chromium platform, Hoechst-stained and PI-negative cells (200,000 cells) from wild-type animals were collected on ice using a cell sorter. The libraries were made using the Chromium platform and Chromium Single Cell 3’ v3 chemistry. Sequencing libraries were loaded on an Illumina nova 6000 flowcells with two 150 bp paired-end kits.

### Spatial transcriptome

Planarian worms were placed at the center of the cryomold and emerged in an ice and water mixture to keep the worm stational before applying chilled cryostat (O.C.T. Compound) embedding media (Sakura, 4583), followed by immediate snap-freeze in isopentane and liquid nitrogen bath. OCT-embedded sample blocks were cryosectioned in a pre-cooled cryostat at 10 µm thickness and placed onto the active capture area of Visium TO and GE slides (10x Genomics, Visium). A permeabilization time of 22 min was determined using the TO slide according to the manufacturer’s instructions. GE slides were fixed and H&E stained before being imaged by Perkinelmer Polaris at 40x magnification and Leica Aperio CS2 at 20x magnification. The tissue was then permeabilized, and reverse transcription and second-strand synthesis were performed, followed by library construction according to the manufacturer’s instructions. Libraries were sequenced on an Illumina Nova platform using a 150 base-pair paired-end dual-indexed setup (High output, v 2.5, Illumina). Each capture area in the GE slide was deep sequenced to the depth of 250,000 mean reads per tissue.

### Whole-mount in situ hybridization

Whole-mount in situ hybridization staining was performed as previously described in Ref. ^42^. Briefly, riboprobes were synthesized using an in vitro transcription kit (Roche, 11277073910) with the addition of digoxigenin/fluorescein-labeling mix (Roche, 11277073910, 11685619910) according to the manufacturer’s instructions. Animals were treated with 5% NAC for 5 min to remove mucus, and fixed in 4% formaldehyde in PBSTx for 30 min at room temperature. Then animals were dehydrated and stored in 100% methanol at −20 °C for at least 1 h. After rehydration, the animals were bleached in formamide-based H_2_O_2_ bleaching solution under bright light for 2 h, followed by proteinase K (Roche, 03508838103) treatment for 10 min, post-fixation and pre-hybridization for 1 h. Hybridization was performed at 56 °C in riboprobe (1:1000) containing hyb buffer for >16 h. After the animals were washed and blocked, they were further incubated in anti-Digoxigenin-POD, Fab fragments (1:2000, Roche, 11207733910; RRID: AB_514500) or anti-Digoxigenin-AP, Fab fragments (1:2000, Roche, 11093274910; RRID: AB_514497) overnight at 4 °C for 16 h, followed by an extensive wash. For colorimetric whole-mount in situ hybridization, BCIP/NBT was used as substrates. For fluorescent development, a tyramide signal amplification system was used. For double-color fluorescence in situ hybridization, peroxidase inactivation was performed between signal developments in 100 mM NaN_3_ for 60 min. The primers used for gene clone and probe template synthesis are listed in Supplementary Data 5. Quantification of fluorescent signals was analyzed by ImageJ software (V1.5) as previously described in Ref. ^43^.

### Whole-mount immunofluorescent staining

Immunofluorescence staining was performed as described before^41^. Different treatments were used for different antibodies. Following antibodies were used: Phospho-histone H3 (Ser10) (H3p) (1:1000, Abcam, ab32107). Planarians were sacrificed in 5% *N*-acetyl cysteine (NAC) for 5 min at room temperature. After being bleached with 6% H_2_O_2_ overnight, animals were washed with PBSTx (0.3% Triton X-100). After 2 h of blocking with 1% BSA, animals were incubated with primary antibody overnight, then washed with PBSTx more than six times and 1 h each. Blocking with 1% BSA for 1 h was performed before the incubation of the fluorescent secondary antibody cy3-labeled goat anti-rabbit IgG (1:1000, Beyotime, A0516) overnight. After extensive wash with PBSTx for more than 6 h, the animals were mounted with 80% glycerol containing Hoechst 33342 (10 µg/ml, Invitrogen, H3570). Quantification of fluorescent signals was analyzed by ImageJ software (V1.5) as previously described^43^.

### TUNEL assay

The TUNEL experiment was performed using ApopTag TUNEL Kit (Millipore, S7165) to stain apoptotic cells following the manufacturer’s instructions. Briefly, animals were treated with 5% NAC (diluted in PBS) to remove the mucus for 5 min with gentle agitation at room temperature, followed by fixation with 4% formaldehyde (0.3% TritonX-100) for 30 min at room temperature. After bleached in 6% H_2_O_2_ (diluted in PBST) in bright light overnight, the animals were incubated with a TdT reaction mixture for 4 h at 37 °C. Next, the samples were washed and blocked before the fluorescent-labeled anti-DIG antibody (1:1000, Millipore, S7165) was added and incubated for 4 h at room temperature. After extensive wash for 4 h in PBSTx, samples were mounted and images were obtained with Leica SP8 confocal microscope. ImageJ was used for the quantification of histological images.

### Processing of spatial transcriptome RNA-seq data

For spatial transcriptome RNA-seq data, reads were processed with the Space Ranger 2.0.0 software from 10X Genomics, aligning and summarizing UMI counts against the *Schmidtea mediterranea* transcriptome for each spot on the Visium spatial transcriptomics array. The files generated from Space Ranger, including raw UMI count matrices, related images, spot coordinates, and scale factors, were imported into R and only retained the spots overlaying tissue sections. Raw counts were normalized using the NormalizeData or sctransform function in *Seurat*^44^.

### Three-dimensional spatial clustering

The three-dimensional reconstructed planarian regeneration models at six time points were aligned based on their shapes, and the centrally located pharynx. After obtaining the three-dimensional relative spatial coordinates, a 3D spatial neighbor network was generated using the Python package *STAGATE*^18^. In brief, spots with a distance of less than 150 in the same section were linked, and those from adjacent sections with a distance of less than 100 were linked. The expression matrix and 3D network were fed into *STAGATE*^18^, and the resulting feature representations were used to extract 3D spatial domains using the R package *mclus*t^45^.

### Processing of scRNA-seq data

For scRNA-seq data, reads were processed with the Cell Ranger 3.0.0^46^ software from 10X Genomics with default and recommended parameters. The sequencing files were aligned to the *Schmidtea mediterranea* transcriptome smed_20140614 using the Cell Ranger based on STAR algorithm^47^. Next, Gene-Barcode matrices were generated for each sample by counting unique molecular identifiers (UMIs) and filtering non-cell associated barcodes. Only genes that can be translated into proteins were retained.

For the *plk1* knockdown sample, we processed the data using a similar way as described above. To identify the cell types of the *plk1* knockdown sample, we used the “ingest” function in *Scanpy*^48^ with the default parameters.

### Single-cell RNA-seq data analysis

Low-quality cells (nGene <500) from the raw sequencing were excluded. To remove the doublets, we excluded cells with excessive reads (nCount >30,000 or nGene >6000), performed scrublet algorithm^49^, and filtered cells with scrublet doublet scores bigger than 0.5. For each cell, UMI counts per gene were normalized to the total UMI count of the cell, and log-transformed as norm = log(UMI + 1). The 3000 most highly variable genes were used for principal component analysis (PCA). The first 50 PCs were used to construct a shared nearest neighbor graph and further generated the two-dimensional UMAP embeddings using the Python package *scanpy*^48^.

We used a consensus clustering approach based on multiple runs to account for the randomness of the Louvain clustering algorithm. Given the fixed resolution parameter as 2, we run Louvain clustering 100 times with different random seeds on the shared nearest neighbor graph, which produced ~60 clusters per run. We then used the outlier-aware DBSCAN algorithm from the Python package *scikit-learn* to obtain the consensus clustering results across the Louvain clustering feature matrix using the hamming distance. We set the epsilon parameter to 0.1 and the min_samples parameter to 100 because the number of clusters is similar to those observed in the multiple Louvain clustering runs. In this setting, cells were clustered into 61 global clusters. Notably, the result of the DBSCAN algorithm contained a few outliers. We trained a random forest classifier based on the PCs of well-categorized cells to classify outliers. We rescued the outlier with a prediction probability >0.5 and removed the remaining. As a result, only about 0.7% of cells (348 cells) were filtered in this step.

### Spatial cell type deconvolution and colocalization analysis

To map major cell types identified by scRNA-seq in the profiled spatial transcriptomics sections, we employed the cell2location package^16^. First, we estimated the reference transcriptomic profiles for each cell type using a negative binomial regression model. Here, we used scRNA-seq data from all regeneration time points and eliminated the effect of sequencing depth by setting the time points as batches. Then, we estimated the abundance of cell types in the spatial transcriptomic data with its default parameter. Briefly, the expected cell abundance for each spot (N_cells_per_location) was set to 30, and the number of training epochs was set to 30,000. The colocalization values were calculated by counting the cell-type pairs that are abundant in the same spot. Only cell types with the top three estimated abundance in each spot were included.

### Cell type imputation by a deep-learning model

We adopted a convolutional neural network to predict cell types in the space between ST spots of the same section and consecutive sections without sequencing information. In the training process, we first inferred cell types for ST spots using cell2location^16^ and Seurat^44^, respectively, and chose 4456 spots from ten sections which were inferred to be the same type by both two algorithms as training spots. After that, we cropped the H&E stained image according to the training spots’ coordinates into 60 × 60-pixel images and labeled them with inferred cell types. Considering the possible imbalance of the numbers of different cell types, we filtered out redundant training spots randomly and trained the model with 1829 spots’ images extracted from sequenced spots with cell type labels. During the training process, fivefold cross-validation are adopted, and 1/5 of the above images were used as the testing dataset on each training process (Fold 1–5). In the predicting process, for predicting cell types in the space between ST spots of the same section, we calculated the coordinates of new spots in space and cropped new spot images. We predicted cell types in new spots with the trained model and adopted an optional section-specific model to avoid batch effect in different sections.

Especially, when predicting cell types in consecutive sections without sequencing information, we used the adjacent section-specific model to generate pseudo labels and added them into the training dataset to retrain the model. Finally, we combined the raw and imputed spot data consisting of 45,231 spots from 20 sections, to obtain a high-resolution cell atlas.

### Tiling spots from consecutive sections

We proposed an algorithm to tile spots from consecutive sections avoiding cover between spots in space. We extracted the spots’ coordinates in the section which is nearest to the ventral and generated the imputed spots between raw adjacent spots. Considering these raw spots and imputed spots as based spot arrays, we project 45,231 spots with scores of cell type annotations which were from 20 sections into the array, and accumulated scores to depict the spatial distribution of different cell types.

### Construct 3D model of planarian

We employed the characteristic features in the H&E staining as the basis to align two sections for 3D reconstruction, such as contour, photoreceptors, pharynx, cephalic ganglions, ventral nerve cord, and genital chamber. By adjusting the position and rotation of the section to be aligned with another section, two sections were superimposed so that each characteristic feature in both sections was overlaid. The resulting parameters of position and rotation of the superimposed section were used as the information for 3D reconstruction. By using obtained spot coordinate arrays and applying rigid transformation to get aligned spot coordinate arrays. Then we draw the cell distribution with aligned coordinates in a single section respectively and reconstructed them in the 3D space by Imaris (Bitplane Company, Zurich, Switzerland).

### Spatial enrichment test of major cell types

To quantify the enrichment of major cell types along the anterior-posterior axis, we performed hypergeometric tests for cell-type marker genes and anteroposterior axis segment-specific genes. First, we divided planarians into ten segments (0 hpa) and seven segments (other time points) by the anterior-posterior axis. Cell-type marker genes and anterior-posterior axis segment-specific genes were identified by the FindAllMarkers function in *Seurat*^44^. Then we used the phyper function in *stats* to perform the enrichment test for each cell type and each anterior-posterior axis segment. *P* value < 0.05 were considered a significant enrichment. The results of the test were visualized via the pheatmap function in the R package pheatmap.

### Inference of cell communications

To infer cell communications between major cell types, we performed the ligand-receptor analysis via the R package RNAMagnet^50^. We first employed the getLigandsReceptors function to get a ligand-receptor database. Then we used the RNAMagnetSignaling function to infer potential communications between major cell types. The communication strength of the ligand-receptor pairs was estimated based on the average expression of the ligands and receptors in the sender and receiver populations, respectively, and the communication strength between two cell populations was estimated by combining the estimated scores of all the ligand-receptor pairs. By setting the threshold, we identified the population pairs with significant communication and identified the ligand-receptor pairs for each specific population pair. Finally, we visualized the communication network via the chordDiagram function in the R package circlize.

The spatial colocalization test of ligand-receptor pairs was performed by the function glm.nb in the R package MASS. The spatial expression of ligands and receptors was used to fit negative binomial generalized linear models. The coefficients of the fitting results were defined as the spatial colocalization degree of ligand-receptor pairs.

### RNA velocity analysis

Spliced and unspliced RNA were counted with the software Cell Ranger 3.0.0^46^ and were used to infer the differentiation dynamics of cell lineages with the software scVelo with its default parameters^51^.

### Gene module analysis

The Python package Hotspot^17^ was used to detect the gene modules of spatial transcriptome data for each time point. First, we imported the raw counts of each spot after filtering by *Seurat*^44^ into Hotspot^17^. Then we used the create_knn_graph function with “weighted_graph=True n_neighbors=30” to compute the K-nearest-neighbors graph and compute per-gene autocorrelations by the compute_autocorrelations function. Finally, we retained the top 6000 genes ordered by correlation to group genes into modules. The score of module-related genes was computed by the AddModuleScore function in *Seurat*^44^ to detect the expression pattern of each module.

### Gene ontology analysis

To enrich the GO annotation of the planarian, we aligned the planarian transcriptome with the uniport database through the blastx program and selected the best comparison result. In addition, we also integrated the existing GO annotation information from Planmine^52^. Based on the comparison results and the associated GO annotations, the GO annotations of the planarians were enriched. Then, gene ontology (GO) analysis was performed using topGO^53^ with all genes as the background.

### Statistics and reproducibility

All statistical analyses of experiments were performed using Prism software. Statistically significant differences between different groups were evaluated by two-sided unpaired Student’s t-test, Hypergeometric test (one-sided), and two-sided Wilcoxon rank sum test. All significant levels are provided with exact *p* value or *p* < 0.0001. Sample sizes were chosen to be similar to previously published data (5–10 animals) for highly penetrant phenotypes^11–13^. Animals were randomly allocated to RNAi treatment conditions and stainings. No data were excluded from the analyses. For RNAi validations after computational screening (Supplementary Data 4), investigators were blinded to allocation during experiments and phenotype assessment. For all H3P imaging and quantitation, investigators were blind to allocation for outcome assessment. For other phenotype characterization after computational screening (*plk1, osr2, nk4, nb3006, nb0353*), the investigators were not blinded to allocation during experiments and outcome assessment. All the experiments of RNAi taken by bright-field images and used for WISH and FISH staining had been repeated three times independently. For HE staining, one planarian worm for each time points was used for the ST experiment.

### Reporting summary

Further information on research design is available in the Nature Portfolio Reporting Summary linked to this article.

## Supplementary information

Supplementary Information

Description of Additional Supplementary Files

Supplementary Data 1

Supplementary Data 2

Supplementary Data 3

Supplementary Data 4

Supplementary Data 5

Supplementary Data 6

Supplementary Data 7

Supplementary Data 8

Reporting Summary

## Data Availability

The raw data generated in this study have been deposited in the Genome Sequence Archive (GSA) under accession number CRA007941 linked to the project PRJCA011425. The H&E images and processed data generated in this study have been deposited in the OMIX database under accession numbers OMIX003867 and OMIX003889. The reference transcriptome (smed_20140614) used in this study is available in the GEO database under accession code GSE72389. Source data are provided with this paper.

## Source data

Source Data

## Code availability

The codes used in this study are available on Github (https://github.com/yangteam/Spatiotemporal-transcriptomic-atlas-of-planarian-regeneration). It is also deposited at Zenodo (https://zenodo.org/record/7875480#.ZEulVOy-tvc).
