# Supplementary material for: Spatiotemporal transcriptomic atlas reveals the dynamic characteristics and key regulators of planarian regeneration
Source: Nat Commun. 2023 Jun 2;14:3205. doi: 10.1038/s41467-023-39016-0 (PMC10238425; doi:10.1038/s41467-023-39016-0)
Supplement: Supplementary file 1 — Supplementary Information [file 41467_2023_39016_MOESM1_ESM.pdf]

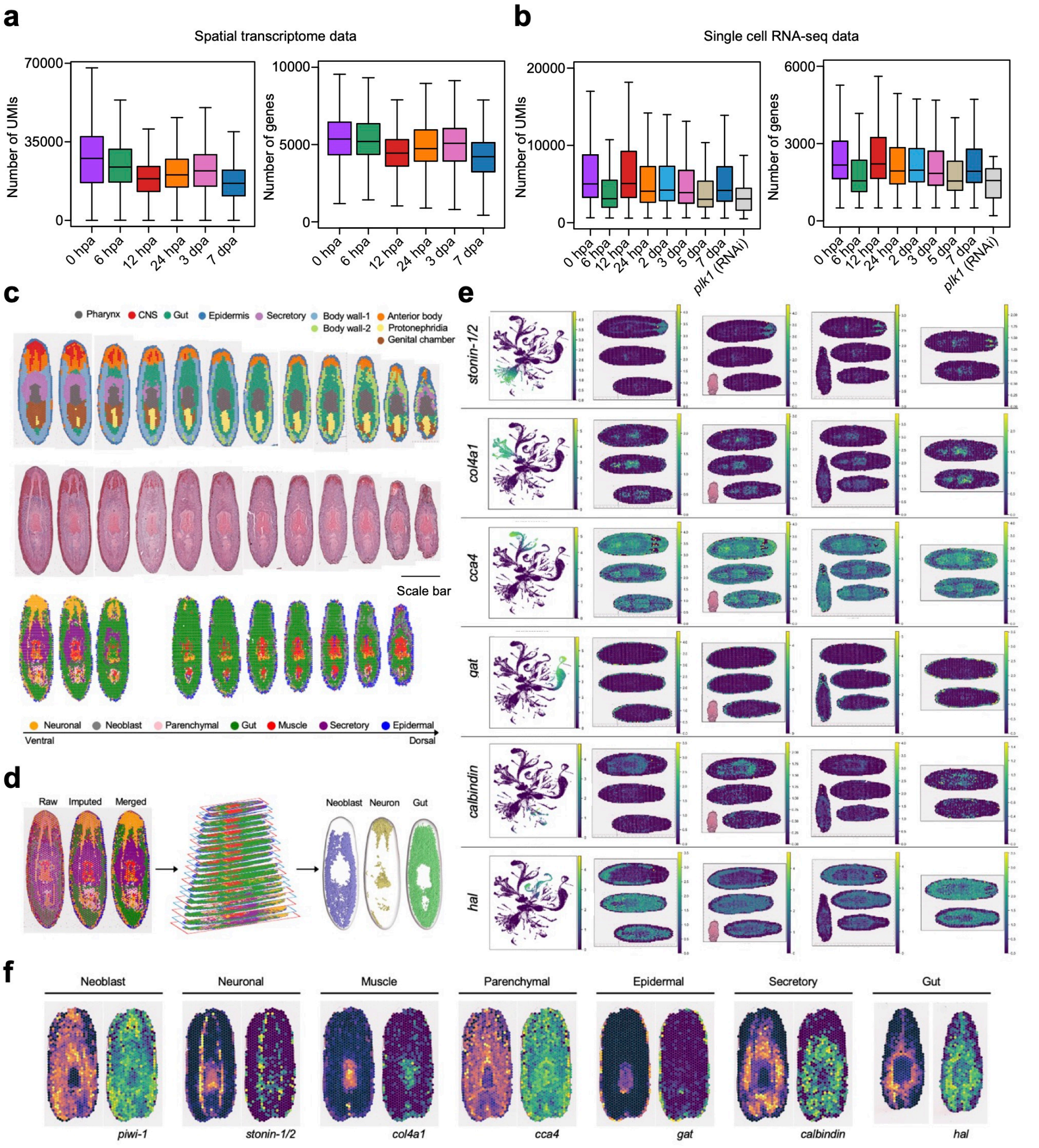

Supplementary Fig. 1. Three-dimensional spatial transcriptomics atlas of planarian *Schmidtea mediterranea*.

(**a**) Boxplot showing the number of UMIs and the number of genes in spatial transcriptome data for each sample. n (from 0 hpa to 7 dpa) = 10101, 7315, 11045, 10612, 9467, 10966. The middle lines of the boxes represent the medians of datasets (50th percentile). The upper and bottom lines of the boxes are respectively the upper quantile (25th percentile) and the lower quantile (75th percentile) of the data. The whiskers mark the upper and lower limits of these datasets.

(**b**) Boxplot showing the number of UMIs and the number of genes in single-cell RNA sequencing data for each sample. n (from 0 hpa to 7 dpa and plk1 RNAi) = 4740, 8924, 5559, 6524, 6017, 8834, 7916, 6500, 7941. The middle lines of the boxes represent the medians of datasets (50th percentile). The upper and bottom lines of the boxes are

respectively the upper quantile (25th percentile) and the lower quantile (75th percentile) of the data. The whiskers mark the upper and lower limits of these datasets.
(**c**) Spatial clustering of ST spots using STAGATE according to gene expression (top). The H&E images (middle) and the cell types (bottom) related to ST data. Scale bar, 2 mm.
(**d**) The workflow of data filling by the machine learning procedure and the virtual model illustrating neoblast, neuron, and gut cells reconstructed.
(**e**) The expression pattern of marker genes for six major types in scRNA-seq data and ST data at 0 hpa.
(**f**) The spatial distribution of major cell types predicted by cell2location and corresponding cell type markers at 6 hpa timepoint. Each panel contains the cell2location result (left) and cell type marker result (right).

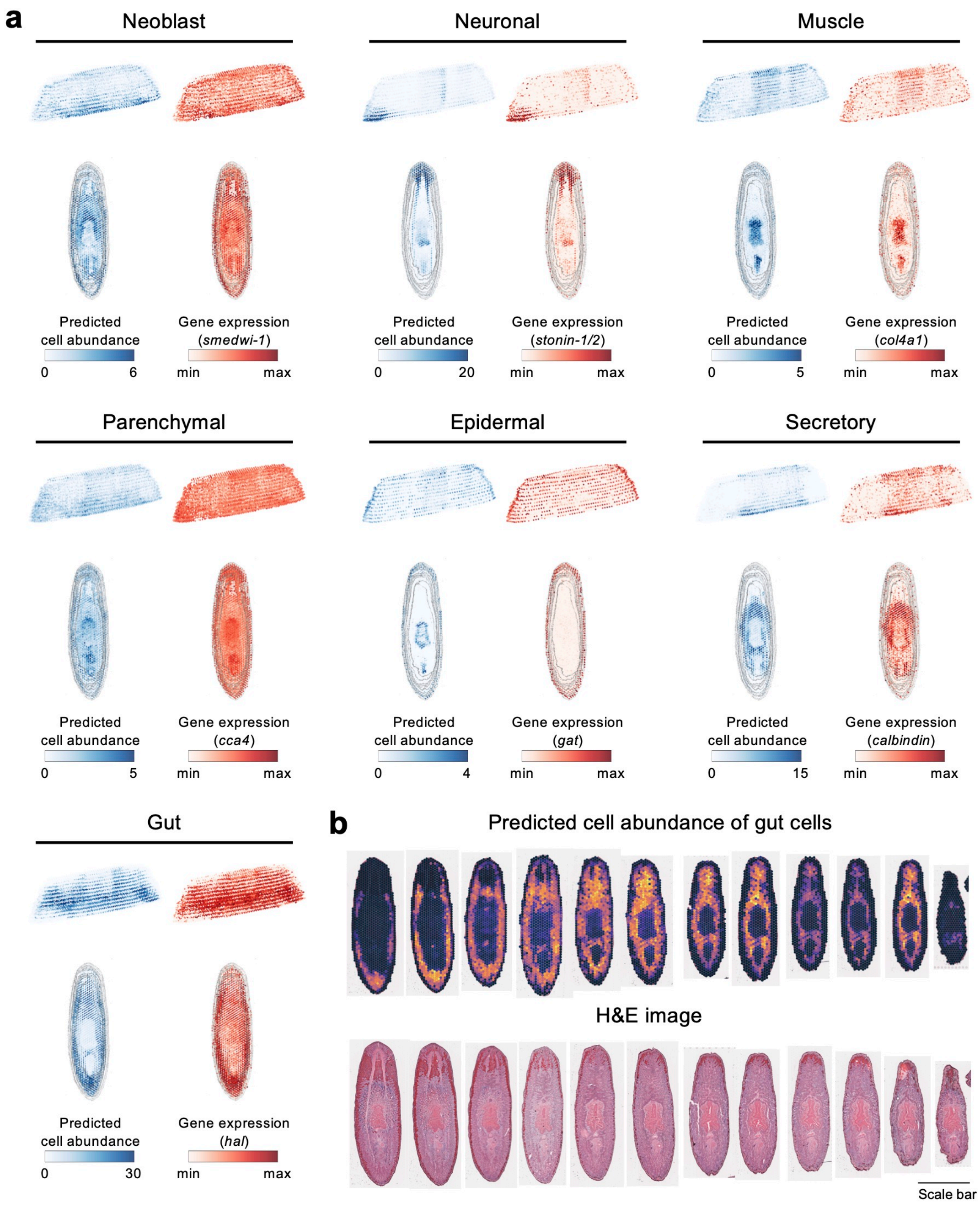

Supplementary Fig. 2. Three-dimensional spatial distribution of cell types in planarian *Schmidtea mediterranea*.

(**a**) The 3D spatial pattern of the deconvolution result for ST spots of each major cell type predicted by cell2location, in comparison to that of each cell marker.

(**b**) Comparison of the spatial distribution of gut cells predicted by cell2location and the H&E staining of the corresponding tissue section. Scale bar, 2 mm.

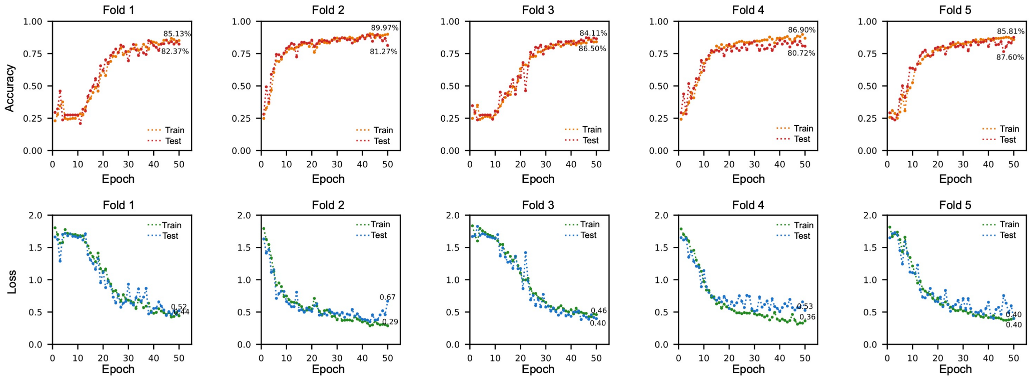

Supplementary Fig. 3. The accuracy and loss of the pretraining model through the 5-fold cross-validation. Source data are provided as a Source Data file.

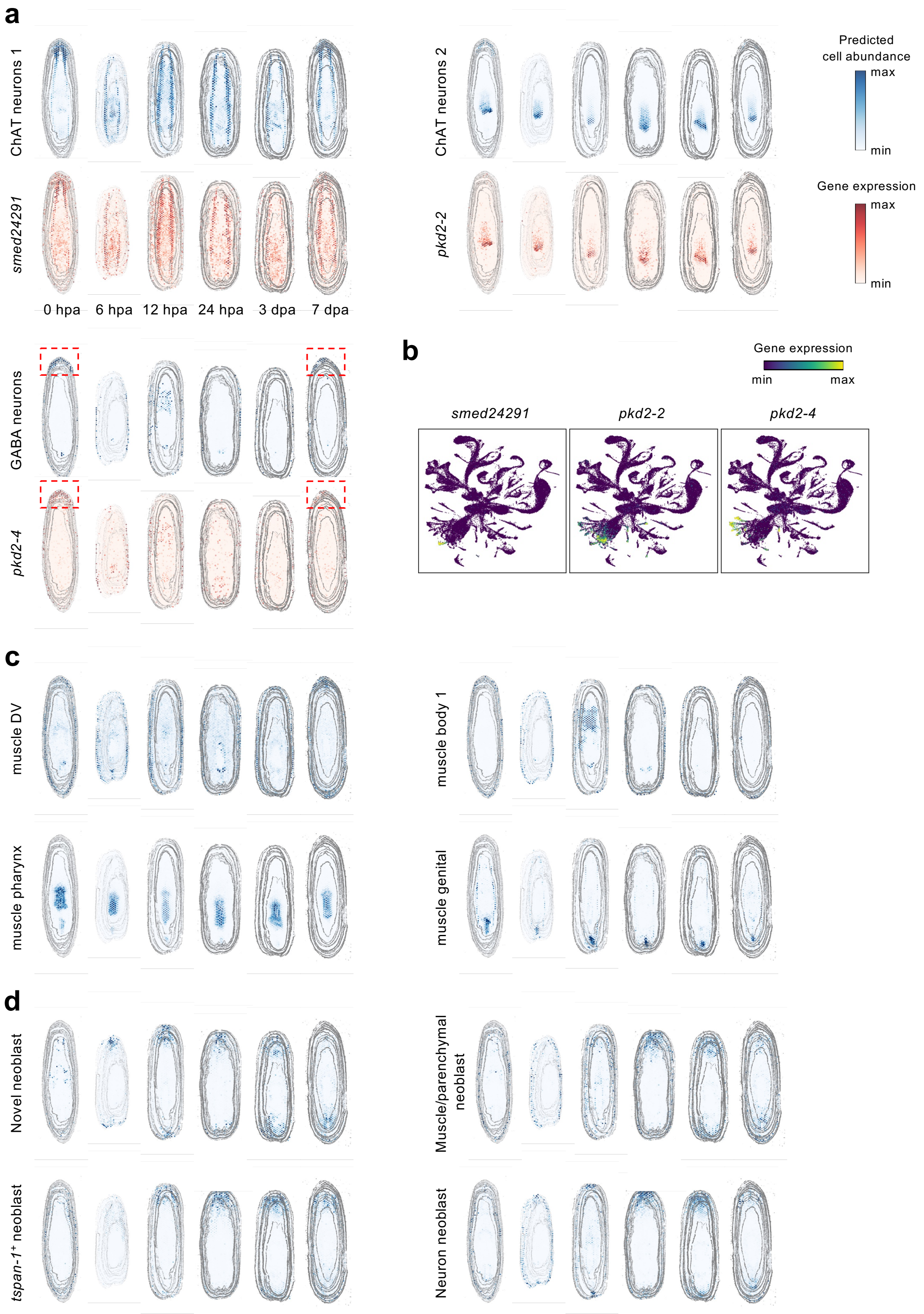

Supplementary Fig. 4. The spatial distribution of subtypes inferred by cell2location.

(**a**) The spatial distribution of three neuronal subtypes and spatial expression of corresponding marker genes.

(**b**) The expression pattern of *smed24291*, *pkd2-2*, and *pkd2-4* in scRNA-seq data.

(**c, d**) The spatial distribution of muscle subtypes (**c**) and neoblast subtypes (**d**).

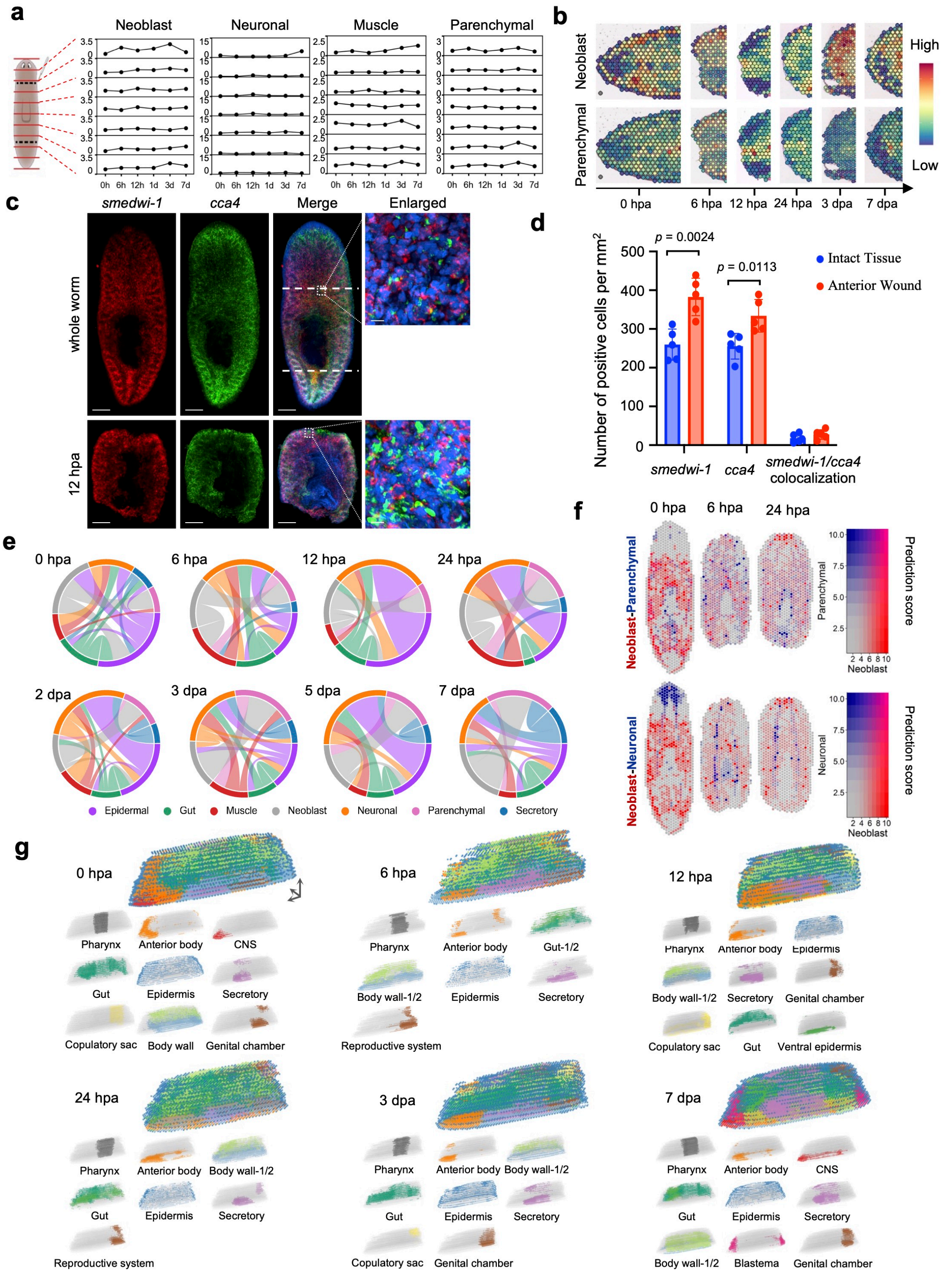

Supplementary Fig. 5. Spatial dynamics of cell types during regeneration.

(**a**) Spatial change of major cell types along the anterior-posterior axis during the regeneration time courses. Source data are provided as a Source Data file.

(**b**) Tissue sections of posterior wound area showing the prediction scores of neoblast and parenchymal of ST spots.

(**c**) FISH staining of neoblast marker *smedwi-1* and parenchymal marker *cca4* in intact planarian and amputated planarian at 12 hpa. Dot line, amputation edge. Scale bar, 300 µm. Enlarged area scale bar, 10 µm.
(**d**) Statistical analysis of number of *smedwi-1*, *cca4* and colocalization of both markers, in wound sites of anterior wound at 12 hpa and corresponding amputation sites in intact planarian. Anterior wound regions, 200 µm from wound edge. Data are the mean ± S.D. (n = 5). The *p* values were determined using a two-sided unpaired Student's *t*-test. Source data are provided as a Source Data file.
(**e**) Chord diagram showing the potential communications between major cell types in all regeneration time points.
(**f**) Co-localization pattern of parenchymal-neoblast (top) and neuronal-neoblast (bottom).
(**g**) Spatial clustering of ST spots using STAGATE according to spatial expression similarity and named by distribution characteristics. In order to compare the features during regeneration, the results of Fig. 1c (0 hpa) is also shown here.

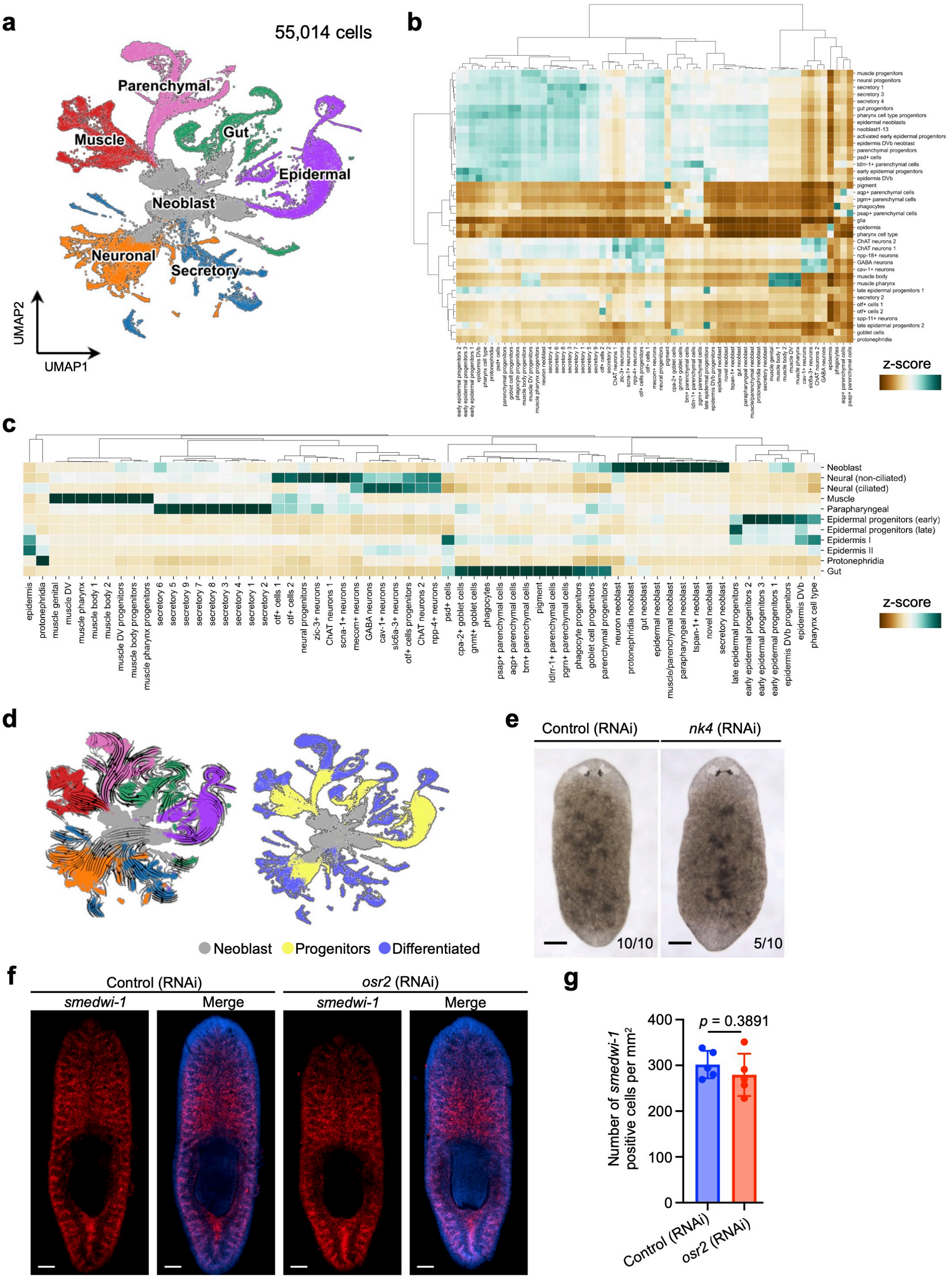

Supplementary Fig. 6. A novel neoblast cell sub-cluster as the most potent stem cell for planarian regeneration.

(**a**) UMAP plot demonstrates main cell types in scRNA-seq data of eight-time points.

(**b**) Correlation of the cell subclusters using previously defined marker genes.

(**c**) Correlation of the major cell clusters using previously defined marker genes.

(**d**) Velocity force field showing the average differentiation trajectories (velocity) for cells located in different parts of the UMAP plot.
(**e**) Bright-field image showing total body sizes for control and *nk4* (RNAi) planarians at 5 dpa. Scale bar, 500 μm. Bottom left number, planarians with the phenotype of total tested. (n = 2 independently experimental replications)
(**f**) FISH staining of neoblast marker *smedwi-1* during homeostasis in control and *osr2* knockdown planarian without amputation.
(**g**) Statistical analysis of the number of *smedwi-1* positive cells per mm2 in (**f**). Error bars represent standard deviation. Data are the mean ± S.D. (n = 5 independent experiments). The *p* values were determined using a two-sided unpaired Student's t-test. Source data are provided as a Source Data file.

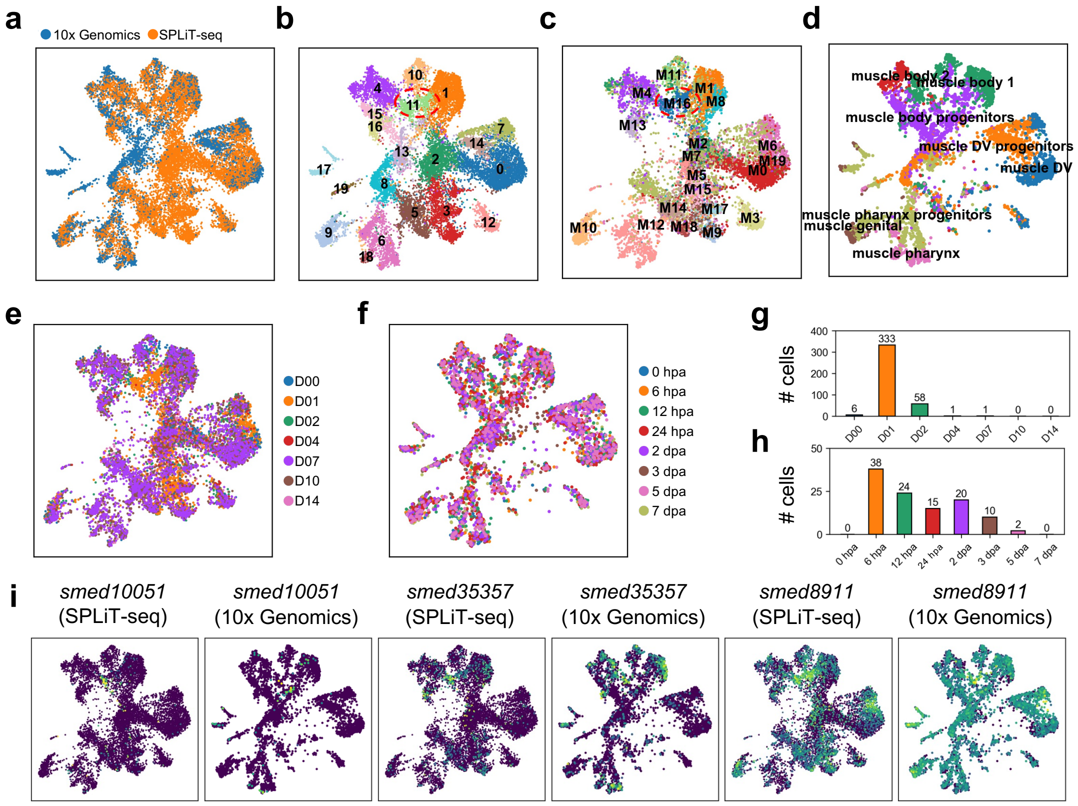

Supplementary Fig. 7. The transient muscle cell type identified by the integration with SPLiT-seq data.

(**a, b**) UMAP plots of the integrated datasets colored by the sequencing technology (**a**) and clusters identified by Louvain algorithm (**b**).

(**c**) UMAP plots of the SPLiT-seq data color by the muscle subtypes identified by Benham-Pyle et al.

(**d**) UMAP plots of the 10x Genomic data generated in this paper colored by the annotations in Fig. 3a.

(**e, f**) The whole-body regeneration time of the SPLiT-seq data (**e**) and the 10x Genomic data (**f**).

(**g, h**) The spatiotemporal distributions of the transient muscle cell type of SPLiT-seq data (**g**) and 10x Genomic data (**h**).

(**i**) The expression pattern of *smed10051*, *smed35357* and *smed8911* in the integrated UMAP space.

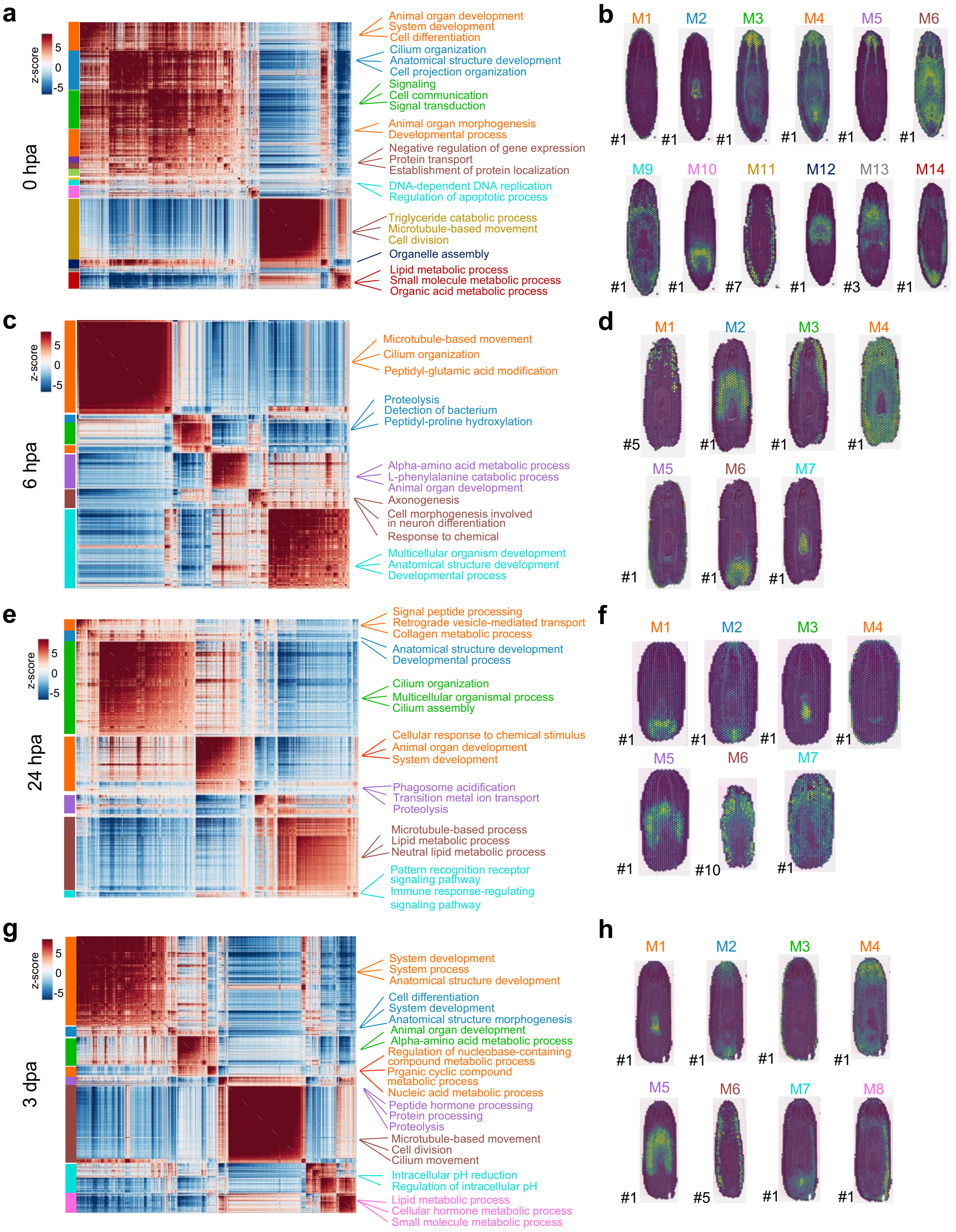

Supplementary Fig. 8. Spatial modules can help to regeneration-related genes.
(**a**) Correlation heatmap of functional gene modules identified by Hotspot analysis in the 0 hpa whole-worm sample. Each row and each column represent a module marker gene, and *Z*-score indicates the correlation between module marker genes. **Right panel:** GO analysis showing the top function terms of each module.
(**b**) The expression pattern of each module identified in (**a**). The ‘‘#number’’ next to the sections represents the number of each section.

(**c**) The gene modules identified by Hotspot analysis in the 6 hpa sample. **Right panel:** GO enrichment analysis showing the top function terms of each module.
(**d**) The expression pattern of each module identified in (**c**).
(**e**) The gene modules identified by Hotspot analysis in 24 hpa sample. **Right panel:** GO analysis showing the top function terms of each module.
(**f**) The expression pattern of each module identified in (**e**). The ‘‘#number’’ next to the sections represents the number of each section.
(**g**) The gene modules identified by Hotspot analysis in 3 dpa sample. **Right panel:** GO enrichment analysis showing the top function terms of each module.
(**h**) The expression pattern of each module identified in (**g**). The ‘‘#number’’ next to the sections represents the number of each section.

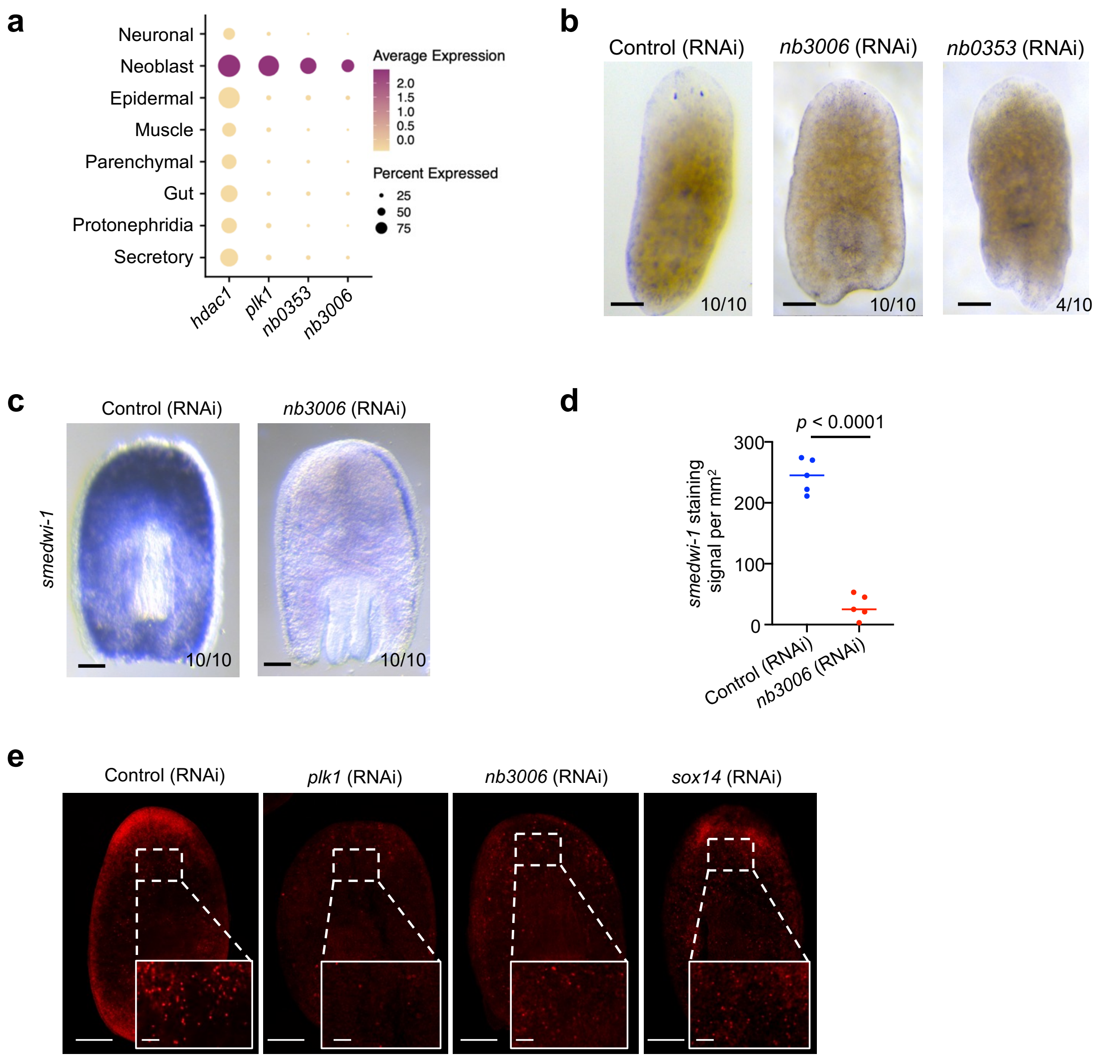

Supplementary Fig. 9. Spatial modules identify key regulatory genes that are essential for regeneration.

(a) Dotplot showing the expression of the hub genes in scRNA-seq data.

(**b**) Bright-field image showing total body sizes for control and *nb3006* (RNAi) and *nb0353* (RNAi) planarians at 5 dpa. Scale bar, 500 μm. Bottom left number, planarians with the phenotype of total tested.

(**c**) WISH staining showing the expression and localization of *smedwi-1* transcripts in control (*control*) and *nb3006* knockdown (*nb3006* RNAi) planarians at 5 dpa. Scale bar, 300 μm. Bottom left number, planarians with the phenotype of total tested.

(**d**) Scatter plot shows the statistical analysis of *smedwi-1* positive cells in control (*control*) and *nb3006* knockdown (*nb3006* RNAi) planarians at 5 dpa. Data are mean ± S.D. and n = 5 animals in each group. The *p* values were determined using a two-sided unpaired Student's t-test (right). Source data are provided as a Source Data file.

(**e**) Immunofluorescence showing the distribution of H3p protein in control, *plk1* (RNAi), *nb3006* (RNAi), and *sox14* (RNAi) planarians at 5 dpa. Scale bar, 300 μm and 50 μm in enlarged field. n (Control) = 8, n (*plk1 RNAi*) = 9, n (*n3006 RNAi*) = 7, n (*sox14* RNAi) = 6.

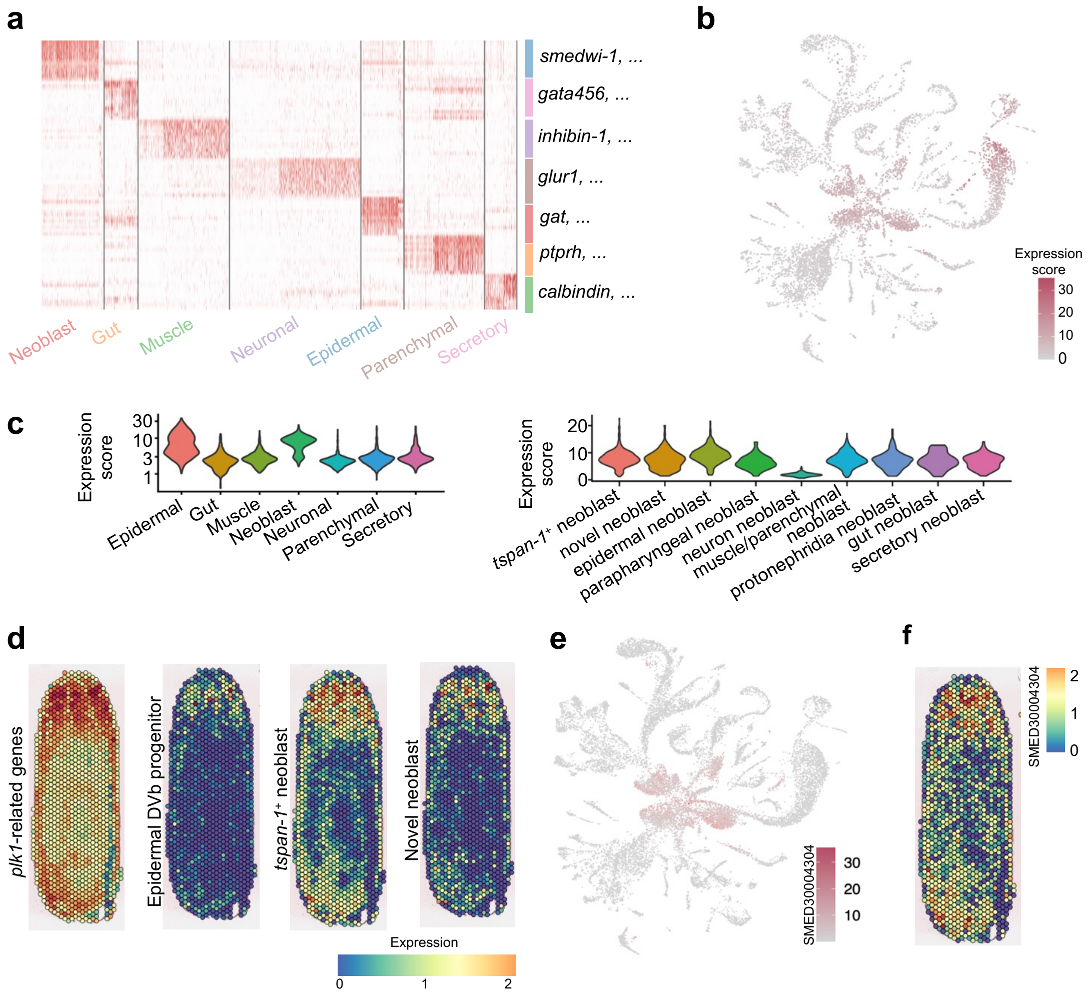

Supplementary Fig. 10. ***plk1*** **is essential for planarian regeneration.**
(**a**) The expression of markers of the major cell types in *plk1* RNAi and control samples.
(**b**) The expression score of the genes that affected by *plk1* and with similar expression pattern to *plk1*, in scRNA-seq data.
(**c**) Violin plot showing the expression score related to (**b**) of major cell types (left), and the subtypes of neoblasts (right).
(**d**) The expression score of *plk1*-related genes and cell types distribution of the first slice related to Fig. 6h.
(**e**) The expression pattern of the gene *smed4304*, which affected by *plk1*, in scRNA-seq data.
(**f**) The expression of *smed4304* in ST data of the first slice at 3 dpa.
