# Supplementary material for: Spatiotemporal transcriptomic atlas reveals the dynamic characteristics and key regulators of planarian regeneration
Source: Nat Commun. 2023 Jun 2;14:3205. doi: 10.1038/s41467-023-39016-0 (PMC10238425; doi:10.1038/s41467-023-39016-0)
Supplement: Supplementary file 2 — Description of Additional Supplementary Files [file 41467_2023_39016_MOESM2_ESM.pdf]

Description of Additional Supplementary Files

File Name: **Supplementary Data 1**
Description: The cell classification of all scRNA-seq data, related to Figure 2 and Figure 6.

File Name: **Supplementary Data 2**
Description: The differentially expressed genes of *osr2*$^{+}$ neoblast cell cluster, related to Figure 3.

File Name: **Supplementary Data 3**
Description: The modules of genes identified by Hotspot for 12 hpa ST data, related to Figure 4.

File Name: **Supplementary Data 4**
Description: List of genes identified in this study that affect regeneration, related to Figure 4 and Figure 5.

File Name: **Supplementary Data 5**
Description: Primers used for dsRNA synthesis, ISH, related to Figure 1-6.

File Name: **Supplementary Data 6**
Description: Summary and statistics for all sequencing data, related to Figure 1, Figure 2 and Figure 6.

File Name: **Supplementary Data 7**
Description: Average cell type scores predicted by cell2location at each section along the AP axis, related to Figure 2.

File Name: **Supplementary Data 8**
Description: The metrics for the model during the training stage, related to Figure 1.
