# Supplementary material for: Spatiotemporal transcriptomic atlas reveals the dynamic characteristics and key regulators of planarian regeneration
Source: Nat Commun. 2023 Jun 2;14:3205. doi: 10.1038/s41467-023-39016-0 (PMC10238425; doi:10.1038/s41467-023-39016-0)
Supplement: Supplementary file 11 — Reporting Summary [file 41467_2023_39016_MOESM11_ESM.pdf]

nature portfolio

Corresponding author(s): Jia-yi Zhou, Jun Cai, Shihua Zhang, Yun-gui Yang

Last updated by author(s): May 3, 2023

Reporting Summary

Nature Portfolio wishes to improve the reproducibility of the work that we publish. This form provides structure for consistency and transparency in reporting. For further information on Nature Portfolio policies, see our Editorial Policies and the Editorial Policy Checklist.

Statistics

For all statistical analyses, confirm that the following items are present in the figure legend, table legend, main text, or Methods section.

n/a	Confirmed	
☐	☒	The exact sample size (*n*) for each experimental group/condition, given as a discrete number and unit of measurement
☐	☒	A statement on whether measurements were taken from distinct samples or whether the same sample was measured repeatedly
☐	☒	The statistical test(s) used AND whether they are one- or two-sided<br>*Only common tests should be described solely by name; describe more complex techniques in the Methods section.*
☒	☐	A description of all covariates tested
☒	☐	A description of any assumptions or corrections, such as tests of normality and adjustment for multiple comparisons
☐	☒	A full description of the statistical parameters including central tendency (e.g. means) or other basic estimates (e.g. regression coefficient) AND variation (e.g. standard deviation) or associated estimates of uncertainty (e.g. confidence intervals)
☐	☒	For null hypothesis testing, the test statistic (e.g. *F*, *t*, *r*) with confidence intervals, effect sizes, degrees of freedom and *P* value noted<br>*Give P values as exact values whenever suitable.*
☒	☐	For Bayesian analysis, information on the choice of priors and Markov chain Monte Carlo settings
☒	☐	For hierarchical and complex designs, identification of the appropriate level for tests and full reporting of outcomes
☐	☒	Estimates of effect sizes (e.g. Cohen's *d*, Pearson's *r*), indicating how they were calculated

Our web collection on statistics for biologists contains articles on many of the points above.

Software and code

Policy information about availability of computer code

Data collection	Image acquisition: Opera Phenix High-content Confocal System and Olympus FV3000RS were used to obtain confocal images of whole-mount immunostaining and in-situ hybridization; PerkinElmer Vectra Polaris and Leica Aperio CS2 were used to obtain H&E images of cryosection for spatial transcripomic sequencing; Leica Stereoscope M5 was used for obtain bright-field images of planarian and colorimetric ISH images.
Data analysis	For spatial transcriptome RNA-seq data, reads were processed with the Space Ranger 2.0.0 software from 10X Genomics, aligning and summarizing UMI counts against the Schmidtea mediterranea transcriptome for each spot on the Visium spatial transcriptomics array. The files generated from Space Ranger, including raw UMI count matrices, related images, spot coordinates, and scale factors were imported into R and only retained the spots overlaying tissue sections. Raw counts were normalized using the NormalizeData or sctransform function in Seurat .<br>For scRNA-seq data, reads were processed with the Cell Ranger 3.0.0 software from 10X Genomics with default and recommended parameters. The sequencing files were aligned to the Schmidtea mediterranea transcriptome smed_20140614 using Cell Ranger based on STAR algorithm (v2.5.1b). Next, Gene-Barcode matrices were generated for each sample by counting unique molecular identifiers (UMIs) and filtering non-cell associated barcodes. Only genes that can be translated into proteins were retained.<br>For the plk1 knockdown sample, we processed the data using a similar way as described above. To identify the cell types of the plk1 knockdown sample, we used the “ingest” function in Scanpy with the default parameters.<br><br>Software, packages and algorithms version are listed here:<br>Space Ranger: 2.0.0<br>Seurat: 4.1<br>STAGATE: 1.0.1

mclust: 6.0.0
Cell Ranger: 3.0.0
STAR: v2.5.1b
Scanpy: 1.9.1
scrublet: 0.2.3
Cell2location: 0.1
Imaris: 8.0.1
ImageJ: 1.53C
pheatmap: 1.0.12
RNAMagnet: 0.1.0
MASS: 7.3.58.1
scVelo: 0.2.4
Monocle: 2.26.0
Hotspot: 0.9.1
topGO: 2.28.0
stats: 4.1.0
Louvain: based on leidenalg 0.8.8
DBSCAN: based on scikit-learn 1.1.2
circlize: 0.4.13

For manuscripts utilizing custom algorithms or software that are central to the research but not yet described in published literature, software must be made available to editors and reviewers. We strongly encourage code deposition in a community repository (e.g. GitHub). See the Nature Portfolio guidelines for submitting code & software for further information.

Data

Policy information about availability of data

All manuscripts must include a data availability statement. This statement should provide the following information, where applicable:

- Accession codes, unique identifiers, or web links for publicly available datasets
- A description of any restrictions on data availability
- For clinical datasets or third party data, please ensure that the statement adheres to our policy

The raw data generated in this study has been deposited in the Genome Sequence Archive (GSA) under accession number CRA007941 (https://ngdc.cncb.ac.cn/gsa/browse/CRA007941) linked to the project PRJCA011425 (https://ngdc.cncb.ac.cn/bioproject/browse/PRJCA011425).
The H&E images and processed data generated in this study has been deposited in the OMIX database under accession number OMIX003867 (https://ngdc.cncb.ac.cn/omix/release/OMIX003867) and OMIX003889 (https://ngdc.cncb.ac.cn/omix/release/OMIX003889). The reference transcriptome (smed_20140614) used in this study is available in the GEO database under accession code GSE72389 (https://www.ncbi.nlm.nih.gov/geo/query/acc.cgi?acc=GSE72389). The Source data are provided with this paper.

Human research participants

Policy information about studies involving human research participants and Sex and Gender in Research.

Reporting on sex and gender	N/A
Population characteristics	N/A
Recruitment	N/A
Ethics oversight	N/A

Note that full information on the approval of the study protocol must also be provided in the manuscript.

Field-specific reporting

Please select the one below that is the best fit for your research. If you are not sure, read the appropriate sections before making your selection.

[x] Life sciences [ ] Behavioural & social sciences [ ] Ecological, evolutionary & environmental sciences

For a reference copy of the document with all sections, see nature.com/documents/nr-reporting-summary-flat.pdf

Life sciences study design

All studies must disclose on these points even when the disclosure is negative.

Sample size	No sample calculations were performed. Sample sizes were chosen to be similar to previously published data (5-10 animals) for highly penetrant phenotypes (Manuscript References 11-13).
Data exclusions	No data were excluded from the analyses
Replication	The spatial transcriptomic and single cell sequencing data presented in the study were a single experiment and were not independently replicated due to resource constraints. Gene expression patterns of spatial and subcluster enriched genes shown by in situ hybridization (Figures 1d, 3e, 3i, 3j, 4f, 6e, 6i; Supplementary Figures 7c,) are representative of more than 5 animals. H3p and TUNEL staining shown by whole-mount immunostaining are representative of more than 5 animals (Figures 5g-h; Supplementary Figure 7e). All RNAi validations were repeated for three times independently. RNAi phenotyping of regeneration (Figures 3o, 5e; Supplementary Figure 5e) were repeated at least three times with similar results.
Randomization	Animals were randomly allocated to RNAi treatment conditions and stainings.
Blinding	For RNAi validations after computational screening (Supplementary Table 4), investigators were blinded to allocation during experiments and phenotype assessment. For all H3P imaging and quantitation, investigators were blind to allocation for outcome assessment. For other phenotype characterization after computational screening (plk1, osr2, nk4, nb3006, nb0353), the investigators were not blinded to allocation during experiments and outcome assessment.

Reporting for specific materials, systems and methods

We require information from authors about some types of materials, experimental systems and methods used in many studies. Here, indicate whether each material, system or method listed is relevant to your study. If you are not sure if a list item applies to your research, read the appropriate section before selecting a response.

Materials & experimental systems

n/a	Involved in the study	
☐	☒	Antibodies
☒	☐	Eukaryotic cell lines
☒	☐	Palaeontology and archaeology
☐	☒	Animals and other organisms
☒	☐	Clinical data
☒	☐	Dual use research of concern

Methods

n/a	Involved in the study	
☒	☐	ChIP-seq
☒	☐	Flow cytometry
☒	☐	MRI-based neuroimaging

Antibodies

Antibodies used	Phospho-histone H3 (Ser10) (H3p) (1:1000, Abcam, ab32107); Anti-Digoxigenin-POD, Fab fragments (1:2000, Roche, 11207733910; RRID: AB_514500) ; Anti-Digoxigenin-AP, Fab fragments (1:2000, Roche, 11093274910; RRID: AB_514497) ; Cy3-labeled Goat Anti-Rabbit IgG (1:1000, Beyotime, A0516)
Validation	Phospho-histone H3 (Ser10) (H3p) (Abcam, ab32107). Validation: Guedelhoefer, O. C. IV & Sánchez Alvarado A. Planarian immobilization, partial irradiation, and tissue transplantation J. Vis. Exp. (2012);<br><br>Anti-Digoxigenin-POD, Fab fragments (1:2000, Roche, 11207733910; RRID: AB_514500) and anti-Digoxigenin-AP, Fab fragments (1:2000, Roche, 11093274910; RRID: AB_514497). Validation: Zeng, A. et al. Prospectively isolated tetraspanin+ neoblasts are adult pluripotent stem cells underlying planaria regeneration. Cell 173, 1593-1608. e1520 (2018)

Animals and other research organisms

Policy information about studies involving animals; ARRIVE guidelines recommended for reporting animal research, and Sex and Gender in Research

Laboratory animals	Asexual CIW4 strain and sexual S2F8b strain of Schmidtea mediterranea were maintained at 20°C in Montjuïch salts as previously described. Animals were fed weekly with homogenized pig liver. The asexual strain of the planarian is agelsss, while the sexual strain was cultured over 6 months before sampled.
Wild animals	No wild animals were used in this study.
Reporting on sex	Sex information were not collected.

Field-collected samples	No filed-collected samples were used in this study.
Ethics oversight	No ethical approval required for planarians.

Note that full information on the approval of the study protocol must also be provided in the manuscript.
